# High-Density Inverted Micellar Intermediates Promote Membrane Fusion of Cationic Liposomes in Drug Delivery

**DOI:** 10.1021/acs.langmuir.5c00659

**Published:** 2025-07-15

**Authors:** Rejhana Kolašinac, Erik Strandberg, Laura Maria Schmitt, Sebastian Jaksch, Sabrina Berkamp, Georg Dreissen, Asma Qdemat, Stephan Förster, Carsten Sachse, Anne S. Ulrich, Rudolf Merkel, Agnes Csiszár

**Affiliations:** † Institute of Biological Information Processing: IBI-2 Mechanobiology, 28334 Forschungszentrum Jülich, 52428 Jülich, Germany; ‡ Institute of Biological Interfaces: IBG-2, 150232 Karlsruhe Institute of Technology, Eggenstein-Leopoldshafen, 76344 Karlsruhe, Germany; § Jülich Centre for Neutron Science: JCNS-1: Neutron Scattering and Biological Matter, Forschungszentrum Jülich, 52425 Jülich, Germany; ∥ European Spallation Source ERIC, SE-221 00 Lund, Sweden; ⊥ Department of Physics and Astronomy, Ångström Laboratory, Uppsala University, SE-751 20 Uppsala, Sweden; # Ernst Ruska-Centre for Microscopy and Spectroscopy with Electrons: ER-C-3: Structural Biology, Forschungszentrum Jülich, 52425 Jülich, Germany; ¶ Jülich Centre for Neutron Science: JCNS-2: Quantum Matter and Collective Phenomena, Forschungszentrum Jülich, 52425 Jülich, Germany

**Keywords:** membrane fusion, cationic liposomes, 2D/3D phase transition, inverted micellar fusion-intermediates (IMI), interlamellar attachments (ILA), drug delivery

## Abstract

Liposomes have become increasingly popular as carriers for pharmaceutically relevant molecules such as nucleic acids, proteins, or anticancer drugs. The bottleneck in delivering such vehicles is their inefficient endosomal uptake by target cells. To bypass endosomal degradation and enhance delivery efficiency, fusogenic liposomes have been developed. They fuse with extraordinary efficiency with the plasma membrane of mammalian cells and deliver their cargo directly into the cell cytoplasm. Here, we set out to decipher the key to membrane fusion and optimize the liposomal composition accordingly. Special focus has been placed on identifying the intrinsic phase properties of these liposomes. Therefore, giant and small cationic liposomes with outstandingly high membrane fusion efficiency were prepared, and their thermal phase behavior was investigated using fluorescence microscopy, solid-state NMR, small-angle neutron scattering (SANS), and cryo-electron microscopy techniques. Our experiments revealed a temperature-dependent phase behavior of those liposomes. At 25 °C and below, mainly a lamellar phase formed without elevated membrane fusion capacity. At the physiological temperature of 37 °C and above, we found high concentrations of inverted micellar intermediates and interlamellar attachments, presumably as precursors of a high-temperature 3D phase, embedded in a lamellar phase. Their structures were resolved by cryo-electron tomography. We believe that the presence of these metastable fusion intermediate structures enables highly efficient fusion with complex biological membranes under physiological conditions, as is necessary in biomedical applications.

## Graphical abstract

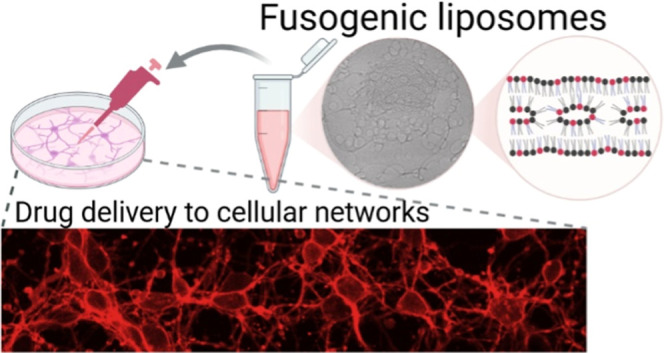

## Introduction

Lipid carriers, such as liposomes or lipid nanoparticles (LNP), are used for the transport of drugs, biomolecules, and imaging agents into living cells.^1–3^ Especially cationic LNPs with small sizes and therefore a high surface-to-volume ratio can encapsulate negatively charged biomacromolecules, such as mRNA or plasmid DNA, and have been successfully used for COVID-19 vaccination in recent years.^4^ The bottleneck of LNP application is the release of cargo molecules from the endosomal pathway into the cytoplasm, from where they can be sorted to the targeted organelle to execute their encoded functions.^5^ Overcoming endosomal uptake is a promising strategy for improving drug delivery success.

Therefore, fusogenic liposomes (FL) were developed.^6^ They are positively charged phospholipid vesicles that have been proven to efficiently deliver membrane dyes,^7^ proteins,^8^ nucleic acids,^9,10^ drugs,^11,12^ or even organelles such as mitochondria^13^ into living cells in vitro and in vivo via membrane fusion. They are based on an equimolar mixture of the neutral phospholipid DOPE and the cationic lipid DOTAP. For molecular structures and membrane architectures, see Figure 1. Even though the two lipids are well-known as fusogenic molecules, especially DOPE,^14,15^ none of them can fuse with biological membranes under physiological conditions (37 °C and pH 7.4) with high efficiency. A significantly increased efficiency, approaching 90%–100%, was achieved by the addition of a third, aromatic compound, such as fluorescent dye molecules,^6^ natural polyphenols,^11^ or some chemotherapeutics.^12^ While fusion with living cell membranes became highly efficient, it still depended very sensitively on liposomal composition. For example, when DOPE was replaced by DOPC, the membrane fusion efficiency of the mixture was significantly reduced.^16^ Instead of fusion, those particles have been internalized via endocytosis by mammalian cells. Changes in liposomal surface charge also influence membrane fusion success; however, a high positive charge alone is no guarantee of high fusion efficiency with biological membranes.^16^

**Figure 1:**
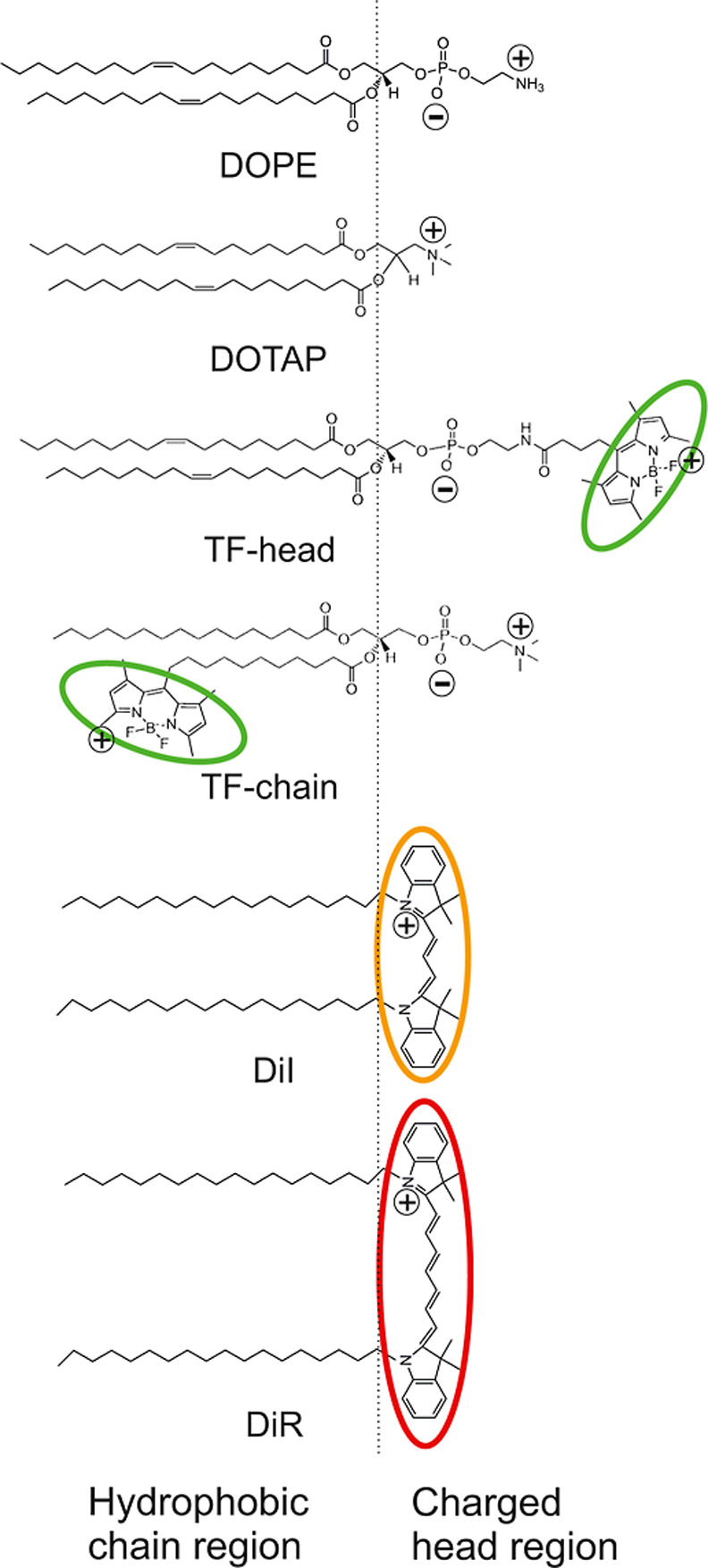
Molecular structures of the fusogenic mixture compounds: the zwitterionic phospholipid DOPE, the cationic DOTAP, the head- and the chain-labeled TopFluor-phospholipids (TF-head and TF chain, respectively), as well as the lipophilic carbocyanine dyes DiI and DiR. The different fluorophores are highlighted by their fluorescence emission colors, and their membrane insertion is indicated. The lipophilic DiI and DiR molecules are fully anchored into the phospholipid membrane by their two long hydrophobic chains, while the aromatic molecular part is close to the phospholipid backbones. The aromatic TopFluor-moiety is stably connected to the membrane surface in the head-labeled phospholipid TF-head. At the same time, it is deeply inserted in the hydrophobic chain region in the case of the chain-labeled lipid TF-chain.

In living cells, membrane fusion is controlled by fusion proteins, e.g., SNARE proteins.^17^ How they mediate fusion has been well studied.^17,18^ Fusogenic liposomes, on the other hand, contain only a few lipids and aromatic molecules without any protein functionalization. To understand the underlying fusion mechanism, systematic material investigations have to be carried out. An earlier study demonstrated that successful fusion depends on the molecular shape of the lipids used, which is typically described by the molecular packing parameter.^16^ These findings indicated that a special “fusogenic phase” is responsible for the observed rapid fusion process. Neutron scattering analysis was performed to determine that lipid phase.^19^ Results suggested a model combining a lamellar phase with micellar inclusions within the usual lipid bilayer. It was proposed that these inclusions caused the favorable fusogenic characteristics. Although these first analyses provided helpful hints, neither the exact lipid phase behavior nor the initial step of the fusion process is sufficiently understood. Here, we tackled these questions with a set of complementary techniques.

In this study, fusogenic liposomes (FL) were prepared using the neutral phospholipid DOPE, the cationic lipid DOTAP, and one of three different aromatic molecules: a carbocyanine dye, and a phospholipid fluorescently labeled either on the head or chain moiety. The molecular structures of the used lipids are shown in Figure 1. When DOPC was used instead of DOPE, liposomes lost their ability to fuse into membranes completely. Such liposomes are typically taken up via endocytosis by mammalian cells; therefore, they will be referred to as endocytic liposomes (EL) and used as control samples.

Initially, a robust protocol was developed to produce giant fusogenic unilamellar vesicles (FL-GUVs) for visualizing the phase behavior and potential phase separation in lipid mixtures. Fluorescence microscopy revealed the formation of domains in micron-sized fusogenic membranes. Therefore, molecular mobility in the different domains was tested with fluorescence recovery after photobleaching (FRAP). In parallel, fusogenic liposomes were imaged on the nanometer scale by cryo-transmission electron microscopy and tomography. Solid-state NMR and small-angle neutron scattering (SANS) techniques were used to identify the fusogenic phase in several lipid mixtures at different temperatures, with a special focus on the body temperature of 37 °C. The combined results shed light on liposomal phase behavior and identified key membrane fusion prerequisites.

## Experimental Section

### Chemicals

All chemicals were used as supplied without further purification. 1,2-dioleoyl-*sn*-glycero3-phosphocholine (DOPC), 1,2-dioleoyl-*sn*-glycero-3-phosphoethanolamine (DOPE), 1,2-dioleoyl-3-trimethylammonium-propane (DOTAP), 1,2-dioleoyl-*sn*-glycero-3-phosphoethanolamine-*N*-[(dipyrrometheneboron difluoride)­butanoyl] (head-labeled TopFluor-DOPE or short TF-head), and 1-palmitoyl-2-(dipyrrometheneboron difluoride)­undecanoyl-*sn*-glycero-3-phosphocholine (chain-labeled TF-DOPC or short TF-chain) were purchased from Avanti Polar Lipids, Inc. (Alabaster, AL, USA). 1,1′-dioctadecyl-3,3,3′,3′-tetramethylindocarbocyanineperchlorate (DiI), and 1,1′-dioctadecyl-3,3,3′,3′-tetramethylindotricarbocyanine-iodid (DiR) were bought from Merck KGaA (Darmstadt, Germany). The used proteins, bovine serum albumin (BSA) and avidin, were also purchased from Merck. Other chemicals such as sucrose, glucose, and phosphate buffered saline (PBS) were obtained from Carl Roth (Karlsruhe, Germany) and Thermo Fisher Scientific (Waltham, MA, USA). Liposomes made of DOPE/DOTAP/X (X = DiR, TF-head, or TF-chain) were used as fusogenic liposomes (FL) while the lipid mixture of DOPC/DOTAP/X was used as nonfusogenic, endocytic liposomes (EL).

### Fusogenic Giant Unilamellar Vesicle Preparation

Giant unilamellar vesicles (GUVs) were produced by the electroswelling technique.^20^ Fusogenic vesicles consisted of DOPE, DOTAP, and DiI or TF-DOPE in a ratio of 1:1:0.1 (mol/mol). Control vesicles were prepared from DOPC and DiI or TF-DOPE in a ratio of 1:0.01 (mol/mol). Lipids were dissolved in chloroform at a concentration of 1 mg/mL, and fluorescent dyes were dissolved at 0.1 mg/mL, and then mixed in the given ratios. Lipid mixtures (15 μL) were spread on nitrogen plasma-cleaned indium tin oxide (ITO) coated glass slides and subsequently dried under vacuum for 15 min. Two slides were then placed in a Teflon chamber, with the coated sides facing each other, and separated by a 1 mm thick Teflon spacer. The chambers were filled with 2 mL of 150 mmol/L sucrose solution, and an alternating current was applied. The swelling parameters for all applied protocols are given in Table S1. Vesicle swelling was repeated at least three times using the same conditions. For quantification, only sedimented GUVs were analyzed after an equilibration time of 30 min at 20 °C.

### Fluorescence Microscopy

#### 2D and 3D Imaging

Plexiglas observation chambers with cover glasses on top and bottom were used to investigate giant vesicles. To avoid vesicle adhesion, the bottom cover glass was coated with BSA for control vesicles or avidin for fusogenic vesicles for 10 min using 1 mg/mL solutions. Excess protein solution was washed off before the GUV solution was carefully added. Vesicles were allowed to sediment for at least 10 min. Imaging was performed using a confocal laser scanning microscope (cLSM710, Carl Zeiss MicroImaging GmbH, Jena, Germany) equipped with an LD-C-Apochromat 63×/1.15 W corr or a 40×/1.1 corr water immersion objective (Carl Zeiss). Vesicles containing DiI were excited using the 543 nm HeNe laser line, and fluorescence was detected between 550 and 750 nm. In comparison, TF-DOPE-containing vesicles were excited with the 488 nm argon laser line, and fluorescence was detected between 500 and 600 nm. Time series were recorded at 20 and 37 °C, and heating was achieved with a standard microscope incubator.

Image analysis was performed using a Python program (version 3.7). First, images of GUVs were smoothed using a Gaussian filter (sigma = 1). Vesicles were detected as circles using the function HoughCircles (OpenCV, method = HOUGH_GRADIENT). At equal angular increments, a total of five times the circle radius (in pixels, size 64 nm) was drawn through the circle center, and the maximum intensity was searched along them in an interval of ±10 pixels around the circle perimeter. From these maximum gray value positions, a mask for the vesicle was created. This mask was then dilated, small holes were filled, and finally skeletonized. For each point on the mask, the gray value and angle, with respect to the circle centroid, were determined.

For 3D imaging, the observation chamber was first filled with 1.5 mL glucose solution with an osmolality of 150 mOsm/kg. Subsequently, 100 μL phosphate buffered saline (PBS, diluted with water to an osmolality of 150 mOsm/kg) was added to the glucose solution to promote weak adhesion of FL-GUVs to the glass surface. Z-stacks were recorded from the bottom to the top of the adhered vesicles. Maximal intensity projections were created using the Arivis extension of the ZEN 2.3 blue lite software (version 2.3.69.1000, Carl Zeiss) and Imaris (version 9.8, Oxford Instruments, Abington, UK).

#### Fluorescence Recovery after Photobleaching

GUVs were seeded and immobilized as described above. 3D images of suitable vesicles were recorded using the same microscope setup as described above. Measurements were performed at room temperature. Circular regions of interest (ROIs) were defined at the vesicle bottom. Where possible, ROIs were chosen on a domain with elevated fluorescence intensity and directly next to the same domain to compare diffusion rates. Laser power was set to 100% for bleaching and 2.5% for recording fluorescence recovery curves. ROIs were bleached, and the average intensity in these regions was recorded over time. The observation interval ranged from 2.4 to 10 s, radii, *r*, ranged from 0.4 to 1.8 μm (average 0.9 μm). Typically, the time increment was 40 ms. Intensities were normalized by dividing them by the average prebleach intensity (*I*_pre_). The time point of half-recovery, *t*_1/2_, was determined by finding the time point at which the intensity was closest to the mean of the intensity directly after bleaching (*I*_0_) and the last measurement point (*I*_∞_). The diffusion constant, *D*, was calculated via^21^

1 $$D=\frac{0.224\times r^{2}}{t_{1/2}}$$

Recovery, *R*, was calculated as

2 $$R=\frac{I_{\infty }-I_{0}}{I_{pre}-I_{0}}$$

Analysis was done in Igor Pro 8 (Wavemetrics, Lake Oswego, OR, USA).

### Cryo-Electron Microscopy

#### Sample Preparation

Vesicles were prepared similarly to those used in SANS experiments and vortexed vigorously before incubation at 4 °C, 22 °C, or 37 °C for 30 minutes. Next, Quantifoil lacey carbon 200 mesh copper grids were glow-discharged, and 3.6 μL of vesicle solution was pipetted onto them. Samples were plunge frozen using a Vitrobot (Mark IV, Thermo Fisher Scientific, Waltham, USA) using a 4 s blot time, −10 blot force, 85% humidity, 4 °C, 22 or 37 °C chamber temperature and vitrified using liquid ethane (Air Liquide GmbH, Germany).

#### Cryo-Electron Microscopy Experiments

Cryo-electron micrographs and tomograms of liposomes were recorded on a 200 kV Talos Arctica G2 electron microscope (Thermo Fisher Scientific, Waltham, USA), equipped with a BioQuantum K3 direct electron detector (Gatan, United States). Zero-loss filtered micrographs were recorded using SerialEM^22^ as movies in super-resolution mode with a pixel size of 0.681 Å and a total dose of 18.18 e/Å^2^, corresponding to 40 frames and a nominal defocus of 5 μm. All tilt series were recorded from −60° to 60° with 3° increments, using a dose-symmetric bidirectional acquisition scheme^23^ with a weighted dose according to the tilt angle. The nominal defocus was set to −5 μm. Raw data were motion-corrected and CTF-corrected using WARP^24^ (version 1.0.9). Tilt series were reconstructed using AreTomo^25^ with a thickness of 3000 pixels at bin 4. To aid in segmentation, a pretrained U-Net model for nondenoised data in MemBrain-Seg^26^ (version 9b) was used, and the output was manually curated in Amira 3D (version 2022.2, Thermo Fisher Scientific, Waltham, USA).^27^ Tomograms were visualized using the IMOD software package (version 4.11.20).^28^

### Solid-State ^31^P NMR

#### Sample Preparation

Vesicle samples for solid-state NMR were prepared using typically 5 mg of other lipids each and 0.5 mg of the fluorescent lipid (total lipid concentration of 10.5 mg/mL). Fluorescent lipids were purchased as chloroform solutions, and the concentration was tested before use. Lipids were purchased as powder, weighed, and dissolved in chloroform. Lipid solutions in chloroform were mixed in a glass vial and dried under vacuum for at least one hour. 150 μL of 10 mM HEPES buffer was added, and the vial was vortexed vigorously, but not sonicated. Samples were placed at −20 °C in a freezer and retrieved after at least one hour, then thawed and vortexed vigorously again. Samples were not heated above room temperature. This freeze–thaw–vortex cycle was repeated five times. Finally, samples were stored at −20 °C. Shortly before the NMR measurements, the sample was removed from the freezer and vortexed one final time. The sample was then transferred to a plastic container and placed in the NMR spectrometer.

#### Solid State NMR Experiments

All NMR measurements were carried out on a Bruker Avance 500 MHz spectrometer (Bruker Biospin, Karlsruhe, Germany). ^31^P NMR measurements were performed on a flat-coil ^31^P/^1^H probe head built in-house using a Hahn echo sequence^29^ with a 90° pulse of 3.5 μs, a 30 μs echo time, and 13 kHz ^1^H SPINAL-64 decoupling^30^ during acquisition. The acquisition time was 10 ms, and the recycle time was 1 s. Typically, 3,000–10,000 scans were collected. For temperature series, an equilibration time of one hour was used between measurements at different temperatures. The sample temperature inside the probe was calibrated using a methanol sample.^31^

^31^P NMR spectra were referenced from a ^1^H NMR spectrum at 30 °C on the same sample, in which the water signal was set to 4.7 ppm, and the corresponding ^31^P reference frequency was calculated from the gyromagnetic ratios of ^1^H and ^31^P.^32^

### Small-Angle Neutron Scattering

#### Sample Preparation

Vesicles were prepared similarly to those used in NMR experiments. The total lipid concentration was set to 20.1 mg/mL. After evaporation of chloroform, the lipid film was resuspended in 20 mM HEPES buffer dissolved in D_2_O (99 atom % D, Sigma-Aldrich, Taufkirchen, Germany) and vortexed vigorously without additional sonification. Samples were stored at −20 °C until usage. One hour before measurements, samples were thawed and vortexed vigorously before being transferred into quartz cuvettes (110-QS, quartz glass, Suprasil, 1 mm path length, Hellma, Müllheim, Germany) for SANS measurements.

#### Small-Angle Neutron Scattering Experiments

Measurements were carried out at the small-angle scattering setup KWS-2, operated by the Jülich Centre of Neutron Science (JCNS) at Forschungsneutronenquelle Heinz Maier-Leibnitz (MLZ), FRM II (Garching, Germany).^33^ A source wavelength of 7 Å (Δ λ/λ = 10%) and a detector system based on an array of ^3^He tubes with a resolution of 8 mm were used for data collection. Sample–detector distances (SDD) of 1.58, 7.58, and 19.48 m were set to cover a *Q*-range of 0.002–0.221 Å^–1^. The exposure time was adjusted to 5, 10, and 20 min for 1.58, 7.58, and 19.48 m SDDs, respectively.

Sample-filled cuvettes were placed in an aluminum holder with a plastic cover. The measurement temperature of 37 °C was maintained by a Peltier element combined with a water bath, controlled by a water thermostat. The scattering intensity of the empty cuvette and the solvent D_2_O were subtracted from the sample scattering. The resulting intensities were azimuthally averaged. All data corrections were performed with the software QtiKWS (JCNS, Jülich, Germany).

#### Model Functions Used for Small-Angle Neutron Scattering Data Fitting

The scattered intensity *I*(*q*) of all data sets was approximated using a linear superposition of scattered intensity of a body-centered-cubic (BCC) lattice made from small vesicles (ca. 25 nm) *I*_BCC_, scattered intensity large vesicles (ca. 500 nm) *I*_ves_ and scattered intensity of a lamellar fraction *I*_lam_ of the sample and incoherent background intensity *I*_B_

3 $$I\left(q\right)={scale}_{BCC}I_{BCC}\left(q\right)+{scale}_{ves}I_{ves}\left(q\right)+{scale}_{lam}I_{lam}\left(q\right)+I_{B}$$

Here, the scale factors are explicitly not volume fractions, as they are not normalized and measure different quantities, such as the total excluded volume, the volume fraction of vesicles, and the volume fraction of lamellae. However, all of them scale with the volume fraction of the corresponding phase, which will be used in this analysis.

The analysis was performed with SASView, while for data analysis, the software SasView (version 4.2.0 (sasview.org)), which also contains the reference to all models presented here.

The scattering from the BCC fraction is described by^34,35^

4 $$I_{BCC}=\frac{V_{lattice}}{V_{p}}P\left(q\right)Z\left(q\right)$$

With *V*_lattice_, the volume of the crystal and *V*_p_ that of the primary single particles, *P*(*q*) the form factor of a simple sphere, and *Z*(*q*) the paracrystalline structure factor for a BCC lattice.

This structure factor is defined as follows in three dimensions

5 $$Z\left(\vec{q}\right)=\prod_{k=1}^{3}Z_{k}\left(\vec{q}\right)$$

with

6 $$Z_{k}\left(\vec{q}\right)=\frac{1-{\left|F_{k}\right|}^{2}}{1-2\left|F_{k}\right|\cos\left(\vec{a_{k}}\vec{q}\right)+{\left|F_{k}\right|}^{2}}$$

and the structure factor of the unit cell

7 $$F_{k}\left(\vec{q}\right)=\exp\left(-\frac{1}{2}\Delta a_{k}^{2}q^{2}\right)\times \exp\left(-i\vec{q}\vec{a_{k}}\right)$$

Here $\vec{a_{k}}$ are the primitive unit cell vectors, and Δ*a*_*k*_ is the isotropic distortion of the lattice point from its ideal position, which allows for the calculation of a distortion factor g=Δa/D , with *D* being the nearest neighbor distance.

The vesicle scattering describes the scattering of large unilamellar vesicles with^36^

8 $$I_{ves}\left(q\right)=\frac{\phi }{V_{shell}}{\left(\frac{3V_{core}\left(\rho_{solvent}-\rho_{shell}\right)j_{1}\left(qR_{core}\right)}{qR_{core}}+\frac{3V_{total}\left(\rho_{solvent}-\rho_{shell}\right)j_{1}\left(qR_{total}\right)}{qR_{total}}\right)}^{2}$$

Here, ϕ is the volume fraction of shell material, *V*_shell_ and *V*_core_ are the volumes of core and shell, respectively, and *V*_total_ is the total volume. The radii of the shell and total vesicle are given by *R*_shell_ and *R*_total_, while the scattering length density of the shell is ρ_shell_ and that of the solvent is ρ_solvent_. *j*_1_ is the first order Bessel function with *j*_1_= (sin *x* – *x*cos *x*)/*x* ^2^. The intensity scattered by lamellae is given by

9 $$I_{lam}\left(q\right)=2\pi \Delta \rho^{2}\Gamma_{m}\frac{P_{bil}\left(q\right)}{q^{2}}Z_{N}\left(q\right)$$

Where the scattering length density contrast is given by Δρ, the mass per unit area bilayer is given by Γ_m_, and *Z*_*N*_ describes the interference within aggregates consisting of more than one bilayer. The bilayer scattering of an infinite planar bilayer of thickness *t* is given by^37^

10 $$P_{bil}\left(q\right)={\left(\frac{\sin\left(qt\right)}{qt}\right)}^{2}$$

The interference term is

11 $$Z_{N}\left(q\right)=\frac{1-w^{2}}{1+w^{2}-2w\cos\left(qD\right)}+x_{N}S_{N}+\left(1-x_{N}\right)S_{N+1}$$

with

12 $$S_{N}\left(q\right)=\frac{a_{N}}{N{\left[1+w^{2}-2w\cos\left(qD\right)\right]}^{2}}$$

and

13 $$a_{N}=4w^{2}-2\left(w^{3}+w\right)\cos\left(qD\right)-4w^{N+2}\cos\left(NqD\right)+2w^{N+3}\cos\left[\left(N-1\right)qD\right]$$

The layer spacing distribution is given by

14 $$w=\exp\left(-\sigma_{D}^{2}q^{2}/2\right)$$

and *D* is the average distance between adjacent layers with a Gaussian distribution with a standard deviation of σ_*D*_.

Because all this appears to be a very large parameter space that would allow for a wide range of possible fitting results, we extrapolated starting values from the TEM images and calculations. In the actual fit, those parameters were left free to show the stability of the fitting result. However, their final values only marginally deviated from those starting values, except for the parameters under investigation, which are the respective scaling factors and the distortion factors of the BCC scattering contribution. Scattering length values were kept constant at 6.36 × 10^–6^ Å^–2^ for the solvent (D_2_O) and 1 × 10^–6^ Å^–2^ for the phospholipids during all fits.

#### Cultivation and Imaging of Rat Embryonal Cortical Neurons

Primary cortical neuronal cells were prepared as described previously by Abraham and co-workers (Animal testing license: 84-02.04.2015.A173, LANUV NRW, Germany).^38^ Isolated cells were cultivated in neurobasal cell culture medium (Thermo Fisher Scientific, Waltham, USA) supplemented with B27 (Thermo Fisher Scientific), Gentamicin (Sigma, Taufkirchen, Germany), and GlutaMAX (Thermo Fisher Scientific). Approximately 30,000 cells/cm^2^ were plated on polylysine-coated (Thermo Fisher Scientific) Petri dishes with glass bottom (ø 1 cm), 3 to 4 days before treatment. Fusogenic liposomes containing DOPE/DOTAP/DiR (1/1/0.1 mol/mol) as well as endocytic liposomes (DOPC/DOTAP/DiR 1/1/0.1 mol/mol) were diluted in PBS at a concentration of 0.1 mg/mL, and the cell culture medium was replaced with the liposomal suspension, and cellular uptake was monitored over time at 37 °C. Live-cell imaging was performed using the same equipment as described above. The fluorescent dye DiR, used for sFL preparation, was excited by the 640 nm HeNe laser line, and the emission signal was detected from 650 nm, applying a 20× Plan Neofluar LD objective (Carl Zeiss).

## Results and Discussion

### Preparation of Fusogenic Giant Unilamellar Vesicles

Giant unilamellar vesicles (GUVs) are convenient tools to investigate thermal phases and phase coexistence in lipid membranes. Generating fusogenic giant unilamellar vesicles (FL-GUVs) presented a challenge. Electroformation, developed by Angelova and Dimitov,^20^ was chosen as the preparation method. This choice avoided contamination of the lipid mixture, which cannot be excluded in oil emulsion or gel-assisted approaches. Control vesicles, made of the neutral monounsaturated phospholipid DOPC, were successfully produced by electroformation using a well-established protocol (see Table S1). Applying the same parameters to the fusogenic lipid mixture, namely the positively charged phospholipid DOTAP, the neutral DOPE, and the head-labeled fluorescent DOPE (TFhead) at a mixing ratio of 1:1:0.1 (mol/mol), gave no GUVs. Therefore, the swelling protocol had to be adjusted.

Nitrogen plasma treatment of the ITO electrodes, increase of AC amplitude and frequency, as suggested by e.g., Shimanouchi et al.,^39^ yielded only very few multivesicular structures that did not sediment. Because temperature can influence GUV formation, we varied this parameter and found that cooling the sample to 3 °C ± 1 °C led to success. With an additionally increased AC amplitude, a sufficient number of GUVs were produced after three hours of swelling (see Tables S1 and S2). The behavior observed for DOPC, i.e., a reduction in diameter with increased AC amplitude and frequency and decreased temperature, agreed with findings from the literature.^40^

### 2D and 3D Imaging of Fusogenic Giant Unilamellar Vesicles

FL-GUVs were imaged by fluorescence microscopy at room temperature. We observed bright spots within the vesicle membrane (Figure 2A). Intensity line profiles along the vesicle membrane (see Figure 2B) showed a sinusoidal pattern with distinct spikes with two times higher intensities compared to control GUVs from DOPC (Figure 2D,E). In addition, no thermal fluctuations were observed in FL-GUVs when heated to 37 °C or subjected to hypo-osmolar conditions as reported earlier,^41^ while DOPC-GUVs displayed strong fluctuations under both conditions.

**Figure 2:**
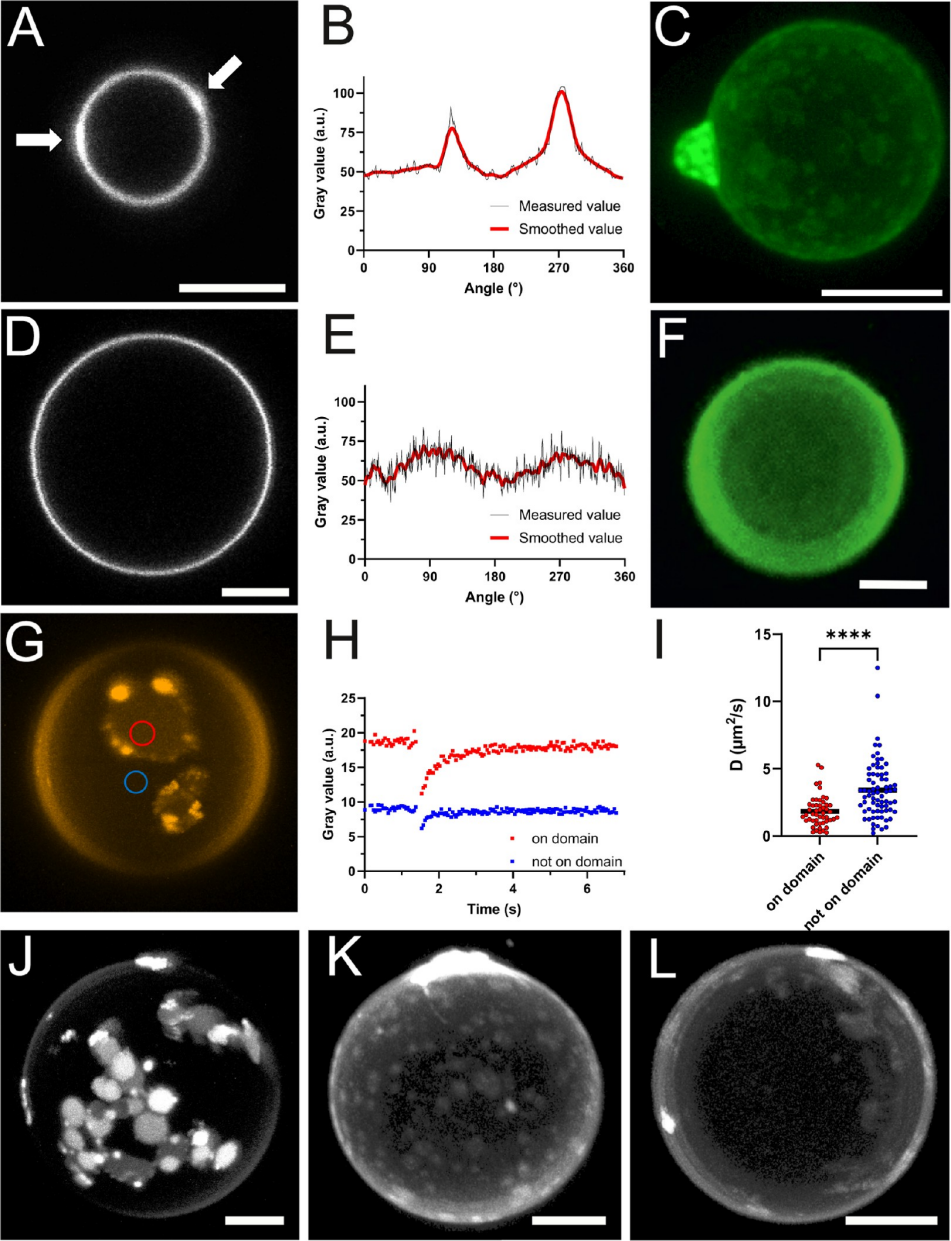
2D and 3D fluorescence imaging of FL-GUVs. (A) Confocal fluorescence micrograph and (B) intensity line profile (angle counterclockwise) of an FL-GUV showing an irregular intensity distribution. Bright intensity spots in the membrane are indicated by white arrows. (C). Bottom-view projection of a 3D scan of an FL-GUV with domain-like lipid segregations. All FL-GUVs contained head-labeled TF-DOPE (TF-head) as the aromatic component. (D) Confocal fluorescence micrograph and (E) intensity line profile (angle counterclockwise) of a control DOPC-GUV with a homogeneous fluorescent intensity distribution. (F) Bottom-view projection of a 3D scan of a DOPC-GUV. (G) Fluorescence micrograph of an FL-GUV containing DiI as the aromatic component. ROI positions of FRAP measurements are indicated by circles. (H) Raw FRAP recovery curves. (I) Distribution of diffusion coefficients *D* measured outside and inside domains. Color code is identical in G, H, and I. Maximum intensity projections of FL-GUVs containing (J) DiI, (K) TF-head, or (L) TF-chain aromatic dyes. All scale bars, 5 μm.

To accurately identify the observed bright spots in fusogenic vesicles, FL-GUVs were immobilized on the glass surface, and z-stacks were recorded using confocal laser scanning microscopy. As presented in Figure 2C,G, 3D-projections of FL-GUVs indicated surface domains enriched in the aromatic fluorophore molecules, while other membrane regions were somewhat depleted. Such lateral phase separation, revealed by an inhomogeneous distribution of fluorophores in the lipid membrane, is a well-known phenomenon. For example, the coexistence of lipid ordered (*L*_o_) and disordered (*L*_d_) phases in phospholipid GUVs containing cholesterol and sphingolipids has been shown by several groups.^42–44^ Later on, their presence has been verified in cellular plasma membrane vesicles as proof for the formation of lipid microdomains, frequently described as lipid rafts, in living organisms.^45^

The observed domain formation in FL-GUVs showed the coexistence of multiple lipid phases. Because the carbocyanine dye DiI is usually enriched in more ordered phases^46^ and thermal fluctuations were absent, it is tempting to speculate about a highly ordered phase.^41^ However, both the round shapes of domains and the high mobility of dye in them (see Figure 2G–I) indicate the absence of crystalline phases. Wang and co-workers have also observed lipid phase separation in DOTAP-containing cationic vesicles. These authors demonstrated that the fusogenicity level of liposomes increased with increasing DOTAP concentration and reported that the simultaneous presence of two phases, liquid ordered (*L*_o_) and liquid disordered (*L*_d_), was a prerequisite for membrane fusion with model GUVs.^47^ However, the coexistence of liquid order and liquid disordered phase neither explains the unusually low area expansion modulus of fusogenic GUVs observed earlier in micropipette aspiration^41^ nor the micellar inclusions observed by small-angle neutron scattering.^19^

Our experiments indicated that the type of fluorophore influenced domain size and shape. DiI-containing FL-GUVs exhibited single circular domains with varying intensities that frequently aggregated on the vesicle surface (Figure 2G,J). The different brightness levels may be attributed to different amounts of the new phase. Unfortunately, its structure cannot be resolved by optical microscopy. When the aromatic molecule was stably connected to the membrane surface using the head-labeled phospholipid DOPE (TF-head), the formed domains resembled rings rather than patches (Figure 2C,K) while aromatic molecules inserted deeply in the hydrophobic chain region, here via a chain-labeled fluorescent lipid (TF-chain), induced very few and amorphous domains with low fluorescence intensity differences compared to the continuous phase (Figure 2L).

As a reference sample, GUVs from DOPC were analyzed. In these, fluorescence was uniform, without any hint of phase separation within the lipid membrane (Figure 2F).

To further investigate the two phases observed in FL-GUVs, molecular diffusion was measured using fluorescence recovery after photobleaching (FRAP) in regions with high and low fluorescence intensity. Circular regions of interest (ROIs) were defined at the bottom of DiI-containing FL-GUVs inside and outside the bright domains, considered as the two different phases (Figure 2G). Exemplary recovery curves are shown in Figure 1H. In some cases, domains with multiple intensity regions were identified. Measurement curves on such an FL-GUV are presented in Figure S1.

In all regions, rapid recovery of bleached fluorophores indicated high molecular mobility. We therefore conclude that all phases involved must be fluid. Diffusion within the bright domains was, on average, slower than in the darker parts. Mean diffusion coefficients (*D*) were 1.8 μm^2^ s^–1^ (s.d. 1.1 μm^2^ s^–1^, *N* = 50 in 39 different vesicles) inside domains and 3.4 μm^2^ s^–1^ (s.d. 2.1 μm^2^ s^–1^, *N* = 73 in 51 different vesicles) outside domains (Figure 2I). It must be noted that measured diffusion coefficients for both phases showed broad distributions with pronounced variation from vesicle to vesicle. In 37 different vesicles, we measured the diffusivity both inside and outside a brighter domain. In 34 of these vesicles, diffusion was slower inside the domain as compared to outside. In only three vesicles, we found equal or faster diffusion inside the domain. Given the scatter of the results, these three diverging observations might be due to noise. Thus, we conclude that diffusion as measured by FRAP in bright domains is slower or at most equal to diffusion in darker background regions. Because measurements had to be performed at the adhered vesicle bottom rather than the top, the obtained diffusion coefficients must be compared to those in supported lipid bilayers. Adhesion to a substrate was shown to slow phospholipid diffusion by a factor of about two in a comparable environment.^48^

Nevertheless, our results can be compared to those of Chiantia and co-workers, who measured the diffusivity of the related dyes DiO and DiD in the *L*_o_ and *L*_d_ domains of supported bilayers formed from a canonical raft mixture (DOPC/sphingomyelin/cholesterol, 1/1/0.67 mol/mol).^49^ While the diffusion constants we measured at a slightly lower temperature were comparable to their results for the *L*_d_ phase, we never observed diffusivities as low as their results for the *L*_o_ phase. Moreover, on average, we observed 1.9 times slower diffusion within domains, while Chiantia et al. report a ratio of 10–20 between *L*_d_ and *L*_o_. Together, these observations argue against an *L*_o_/*L*_d_ coexistence in our samples.

### Imaging of Small Fusogenic Liposomes by Cryogenic Transmission Electron Microscopy and Tomography

Light microscopy resolution was insufficient to distinguish between the two phases that appeared in FL-GUVs with high and low fluorescence intensity. To visualize the phase coexistence in FL samples, electron microscopy with nanometer resolution was employed. To pinpoint a potential thermotropic phase behavior of the fusogenic mixtures, samples were vitrified at temperatures of 4 °C, 20 °C, and 37 °C. Additionally, instead of giant vesicles, small fusogenic liposomes (sFL) were prepared.

As shown in Figure 3A,B, at 4 °C, fusogenic liposomes adopted a 2D lamellar phase with characteristic appearance of uni- and multilamellar vesicles with diameters between 50 nm and larger than 1 μm, and a membrane thickness of 4–6 nm.

**Figure 3:**
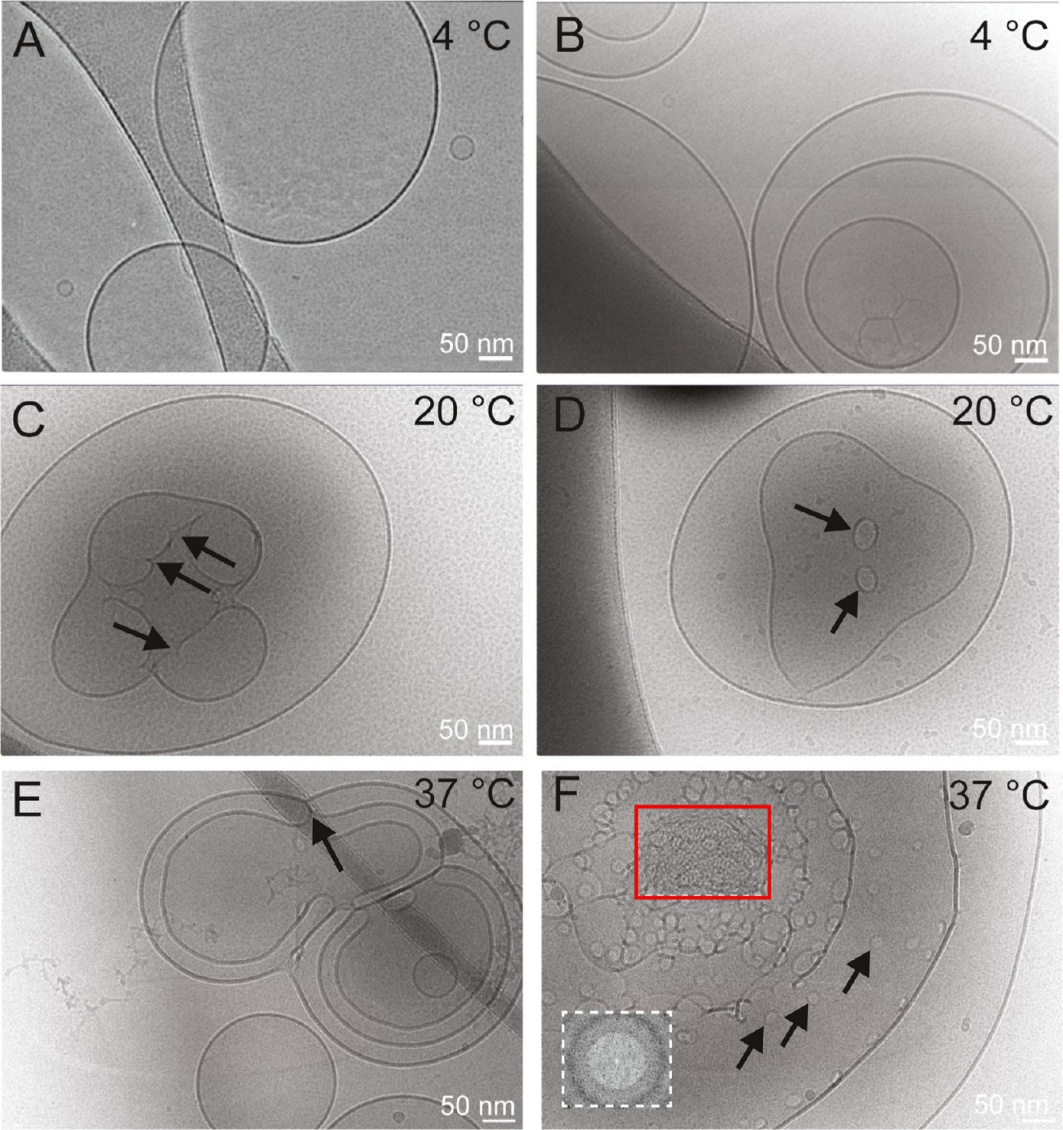
Cryo-TEM visualization of FLs. Phase coexistence formed in sFL samples (DOPE/DOTAP/DiR 1/1/0.1 mol/mol) at 4 °C (A,,B), 20 °C (C,D), and 37 °C (E,F). Arrows denote characteristic patterns such as interlamellar attachments (ILA). Inverted micellar intermediates (IMI) with diameters of 4–8 nm are highlighted by a red square, and their Fourier transform (FT) is shown in the lower left corner in a white square.

At room temperature (20 °C), two additional phase patterns were observed: one with irregular indentations on the membrane surfaces (Figure 3C) and another with spherical structures with diameters between 10 and 50 nm (Figure 3D). We believe that the visualized structures correspond to interlamellar attachments (ILA) similar to those identified by Siegel and co-workers.^50^ While Figure 3C shows bilayer attachments between opposed planar layers from the side view, Figure 3D presents toroidal membrane perforations viewed from upside down on the membrane surface.

At physiological temperature (37 °C), many more ILAs were observed (Figure 3E,F). Moreover, some of those structures had a dense core in addition to the presence of ILAs (Figure 3F, red square). These structures, made of spherical objects with diameters of approximately 4–8 nm, can be interpreted as inverted micellar intermediates (IMI) and initial nucleations of vesicle-to-sponge or to-hexagonal or cubic phases. However, fast Fourier transformation (FFT) analysis did not show any characteristic pattern for those 3D phases.

All other investigated sFL samples showed similar phase coexistences. For example, sFLs containing TF-chain as aromatic molecule exhibited a lamellar phase at 20 °C, while at about 37 °C, altering multiple vesicular structures, identified as IMI and ILAs, were recorded (Figure S2).

Because the micrograph presented in Figure 3F shows a 2D projection image of a 3D object, we sought clarification of the shape of this fusogenic phase. A ±60° tilt series of images was recorded to generate a 3D tomogram, albeit they suffer from the so-called missing wedge.^51^ Similar to cryo-TEM imaging, cryo-electron tomography, followed by volume segmentation of the observed lipid structures, confirmed lamellar phase formation of sFLs with sheet-like bilayers of 4 nm thickness at 20 °C (Figure 4A,B and Movie S1). Compared to room temperature, altered multiple vesicular structures were also observed at 37 °C (Figure 4C,D, and Movie S2). Interestingly, we observed spherical vesicles with diameters of 100–130 nm, composed of single lipid bilayers (thickness approximately 4 nm), as well as dense, undulating membrane segments (thicknesses approximately 9 or 14 nm) close to each other. Remarkably, elongated channels of hexagonal symmetry, which are the hallmark of H_II_ phase formation, were never observed. The structure shown in Figure 4C resembles that of a sponge phase, including central bilayer vesicles that form a complicated meshwork of high-curvature membranes.

**Figure 4:**
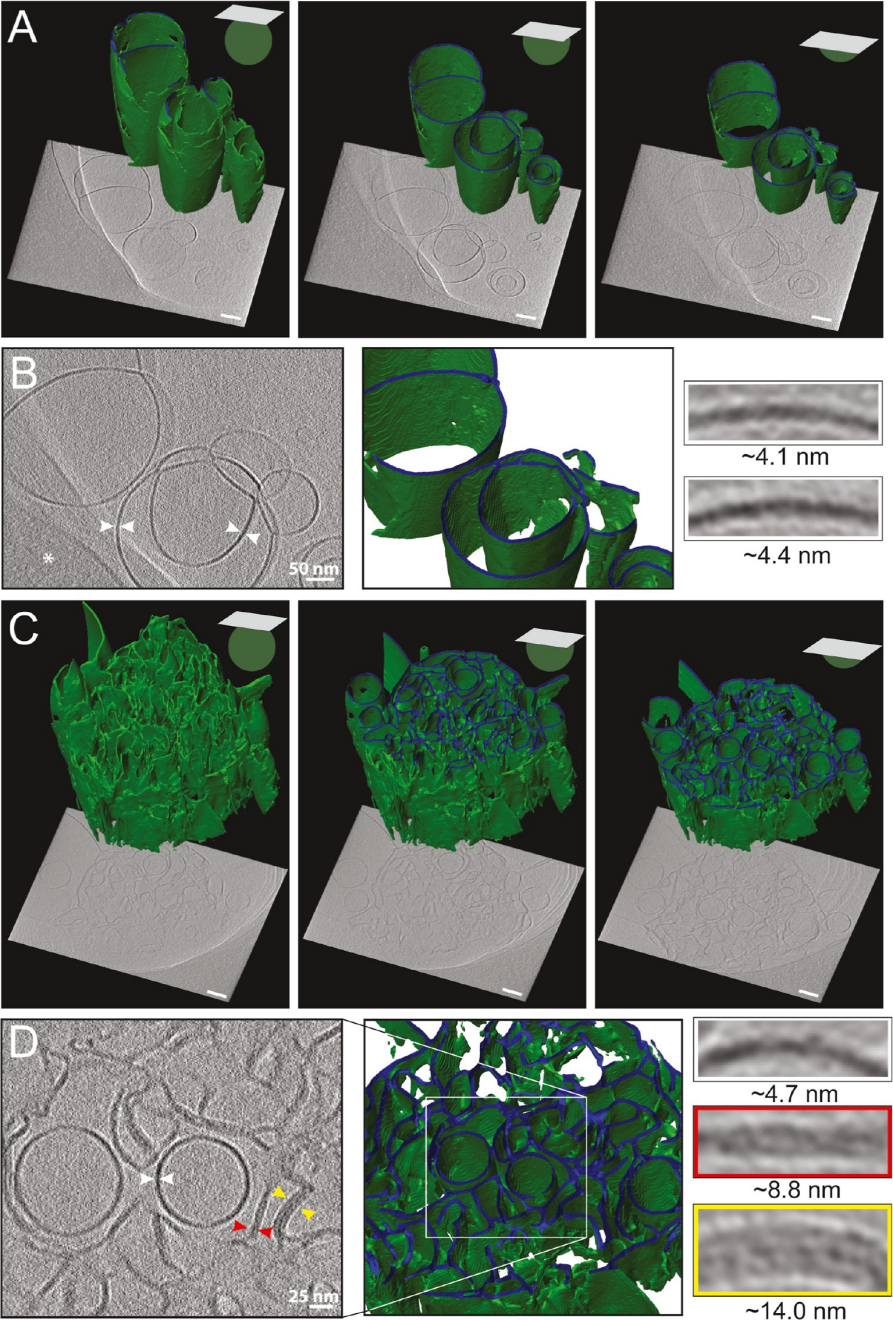
Slices through cryo-electron tomograms and 3D segmentations (green) of sFL structures. The vitrified fusogenic mixtures of DOPE/DOTAP/DiR 1/1/0.1 mol/mol were visualized after incubation at 20 °C (A,B) and 37 °C (C,D), respectively. White arrowheads indicate single, red double-, and yellow triple-bilayers. Z-slices of 3D reconstructions are shown in three distinct planes. The asterisk in panel B marks the edge of the carbon support film that is not part of the sFL. Highlighted membrane bilayers were low-pass filtered to 4 pixels. Scale bars, 100 nm, if not indicated otherwise.

### Lipid Phase Analysis of Small Fusogenic Liposomes by Solid State ^31^P NMR

To identify both phases that appeared in FL-GUVs with high and low fluorescence intensity, and in sFL samples as lamellar and vesicular phases, solid-state ^31^P nuclear magnetic resonance (ssNMR) spectroscopy measurements were performed. We analyzed the temperature-dependent phase behavior of the same three fusogenic mixtures previously described.

Because DOPE alone is known as a fusion-inducing lipid, we also tested its thermal phase behavior. As expected, we observed typical NMR spectra for the inverted hexagonal (H_II_) phase, shown in Figure 5, with a peak maximum close to 5 ppm and a broad shoulder on the right side, in good agreement with the literature.^52,53^ When the cationic lipid DOTAP was added to DOPE at a 1/1 mol/mol ratio, a single lamellar phase (*L*_α_) was observed with a typical highest peak position at −20 ppm and a broad shoulder to 50 ppm. Both phases remained unchanged over the investigated temperature range from 4 to 60 °C (Figure 5).

**Figure 5:**
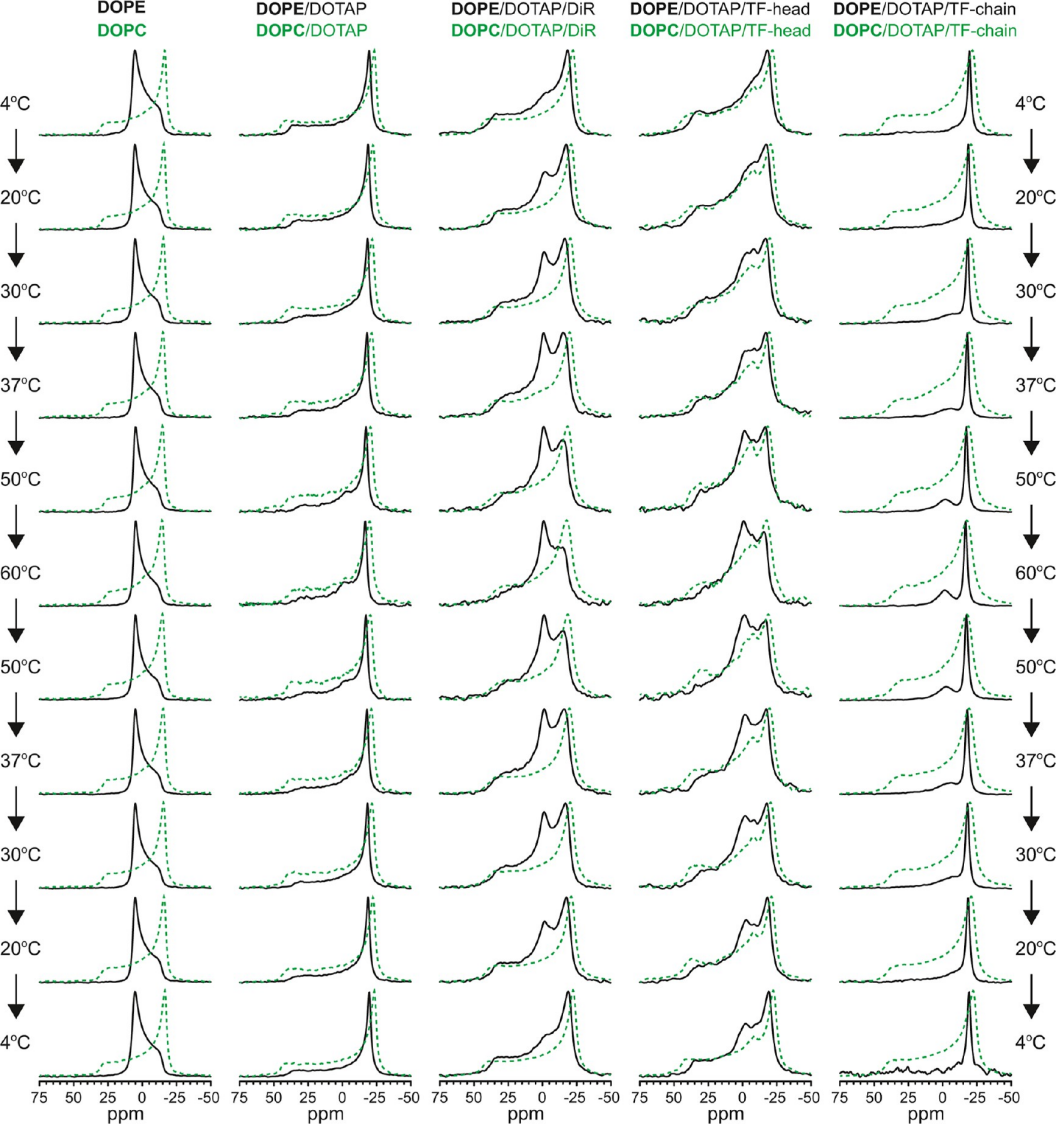
Solid-state ^31^P NMR spectra of sFLs and sELs. DOPE, or DOPE/DOTAP (1/1) with and without the fluorescent lipid analog DiR, the head-labeled (TF-head) or the chain-labeled (TF-chain) phospholipids were investigated. The temperature was first increased from 4 to 60 °C and then decreased again to 4 °C to test reversibility. Only with the TF-head, the isotropic peak was not fully reversible. As control sEL samples, DOPC and DOPC/DOTAP (1/1) with and without the same fluorescent dyes were used (green dotted lines).

To understand the described phase behavior of DOPE alone and in mixtures with DOTAP, the molecular shape of the lipid compounds has to be taken into consideration. For example, because of the small area requirement of the ethanolamine (PE) head and the large area requirement of the two long unsaturated chains (C18:1), DOPE has an inverted conical molecular shape. For such molecules, it is energetically unfavorable to form lamellar bilayers. Instead, they prefer highly curved 3D structures such as inverted hexagonal or cubic phases.^54^ When a molecule with a cylindrical shape, e.g., DOTAP, is mixed with DOPE, the appearance of a lamellar phase is to be expected.^55^ Lipids with equal area requirements in both molecular regions, e.g., DOPC and DOTAP, prefer packing in 2D lamellar bilayers (Figure 5, green line). Membranes with a high content of inverted conical and/or charged lipids are relatively unstable. Therefore, the addition of a third compound can completely change the phase equilibrium.

When an aromatic compound was added to the DOPE/DOTAP mixture, NMR spectra showed the presence of a lamellar phase at up to 20 °C. With increasing temperature, an additional peak at 0 ppm appeared, indicating the formation of a phase with isotropic characteristics. Such a peak could result, for example, from small spherical objects like micelles or inverted micelles, or cubic phases, where the orientation of the ^31^P spin is isotropically averaged by dynamics that are fast on the NMR time scale.^56^ In the case of sFLs containing DiR as aromatic molecules, the highest isotropic peak intensity was higher than the highest lamellar peak intensity. The two peak intensities were approximately equal when a head-labeled lipid (TF-head) was added to the DOPE/DOTAP mixture. Still, only a shoulder remained when the aromatic compound was inserted entirely in the hydrophobic chain region (TF-chain). Repeated experiments on independently prepared samples yielded different intensities of the isotropic peak, as exemplified in Figure S3. The phase transition was reversible with temperature in all cases.

Different aromatic molecules induced different amounts of the nonlamellar phase. On first glance, this phenomenon could be explained by the effective molecular shapes of those molecules, which are usually the most crucial parameter influencing lipid phase formation. The chain labeled lipid, TF-chain, has an inverted-conical shape with the highest potential to induce 3D phase formation. At the same time, the cylindrical TF-head and the carbocyanin dye DiR should be stably embedded into the lamellar phase. However, our experiments using ssNMR and microscopy contradict this hypothesis, specifically that TF-chains with an inverted-conical molecular shape induce noticeably less isotropic phase formation compared to cylindrical molecules.

Presumably, molecular shape is not the only parameter influencing the phase behavior of those mixtures. We hypothesize that an attractive electrostatic interaction between the π-electrons of the aromatic group and the positively charged molecular parts of DOTAP and DOPE guides the phase equilibrium if the molecular counterparts are close to each other. In the case of the head-labeled lipid (TF-head) and the DiR molecules, the aromatic molecular parts are embedded in the lipid headgroup and the lipid backbone membrane regions, close to the positively charged molecular parts of DOTAP and DOPE. In contrast, the aromatic group of the chain-labeled lipid is embedded in the hydrophobic lipid core, which increases the distance and thus substantially reduces interaction strength. As a consequence, a decrease in the isotropic phase formation occurs. We believe that the polarizability of those π-electrons via permanent cations is a prerequisite for fusion induction.

When the aromatic molecule was replaced by a cyclic aliphatic one, e.g. biotin, where all binding electrons are localized on distinct σ molecular orbitals, the fusion potential of sFLs was abolished entirely, as was shown by Kolasinac et al.^16^ Therefore, we investigated the thermal phase behavior of that lipid mixture and mainly found the spectral pattern of a lamellar phase. Only at increased temperature did a relatively flat shoulder appear at 0 ppm (Figure S4). Moreover, the replacement of phosphoethanolamine with phosphocholine (DOPC) results in minimal fusion efficiency,^16^ while the liposomal surface charge remains unchanged. For this mixture, we again found negligible formation of the isotropic phase (Figure 5, green lines). Here, first, phosphocholine is a zwitterionic lipid with a cylindrical molecular shape, preventing the formation of phases displaying high local curvature. Second, the large choline headgroup inhibits electrostatic interactions between the molecular partners within the lipid bilayer, e.g., cationic DOTAP and the π-electrons of the aromatic dyes. These two synergistic effects resulted in significantly reduced membrane fusion efficacy.

### Lipid Phase Analysis of Small Fusogenic Liposomes by Small-Angle Neutron Scattering

Based on the results obtained from ssNMR spectroscopy, it is assumed that all cationic liposomes containing aromatic molecules exhibit a coexistence of a lamellar phase and one that appears isotropic on the time scale of NMR results. Our electron microscopy results further support this. To identify the structure of the newly formed phase, temperature-dependent small-angle neutron scattering (SANS) experiments were conducted in the q-range from 2 × 10^–3^ to 3 × 10^–1^ Å^–1^. The total lipid concentration was increased to 20 mg/mL for efficient signal detection. Because our previous results showed the presence of a lamellar and probably a cubic phase as well as spherical structures, the scattered intensity of all data sets was approximated using the linear superposition of the scattered intensity of a lamellar fraction, a body-centered-cubic (BCC) lattice made from small vesicles (ca. 25 nm), large vesicles (ca. 500 nm), and incoherent background intensity (see eq 3). The lamellar distance, vesicle sizes, and their distances for the BCC phase were taken from TEM images and used to calculate the starting values for fitting, given in Table 1.

**Table 1:** Starting Parameters Used During SANS Data Fitting as Found in TEM Images

Lamellar thickness (nm)	BCC vesicle size (nm)	Nearest neighbor distance (nm)	Large vesicle radius (nm)
3	25	45	500

Scattering patterns of sFL samples are shown in Figure 6. DiR and TF-chain containing sFL exhibited a prominent shoulder at *q* = 0.01 Å^–1^, while sFL containing TF-head as aromatic molecule, displayed peaks at 0.026 Å^–1^ and 0.044 Å^–1^ (Figure 6B). The observed shoulder in the first two samples indicated the presence of large-scale structures, such as vesicles. The appearance of peaks at higher *Q* values in the third sample was attributed to an increase in the BCC domain. Here, it should be noted that those peaks are either identical or in very close proximity to the lamellar peaks from the bilayer distance. Since the nearest neighbor of the BCC and the lamellar distance are very close in all fits, some peak positions are nearly identical (BCC lattice constant ca. 46 nm, first maximum ca. *Q*_1_ = 0.02 Å^–1^, second maximum ca. *Q*_2_ = 0.027 Å^–1^, vesicle bilayer distance ca. 25 nm, first maximum ca. *Q* = 0.025 Å^–1^). It is possible to force the fits in either direction; however, we found the presented values of relative scaling to be stable, while the inverted (higher BCC scaling values exchanged with higher lamellar scaling values) approaches were unstable, resulting in very large errors for both.

**Figure 6:**
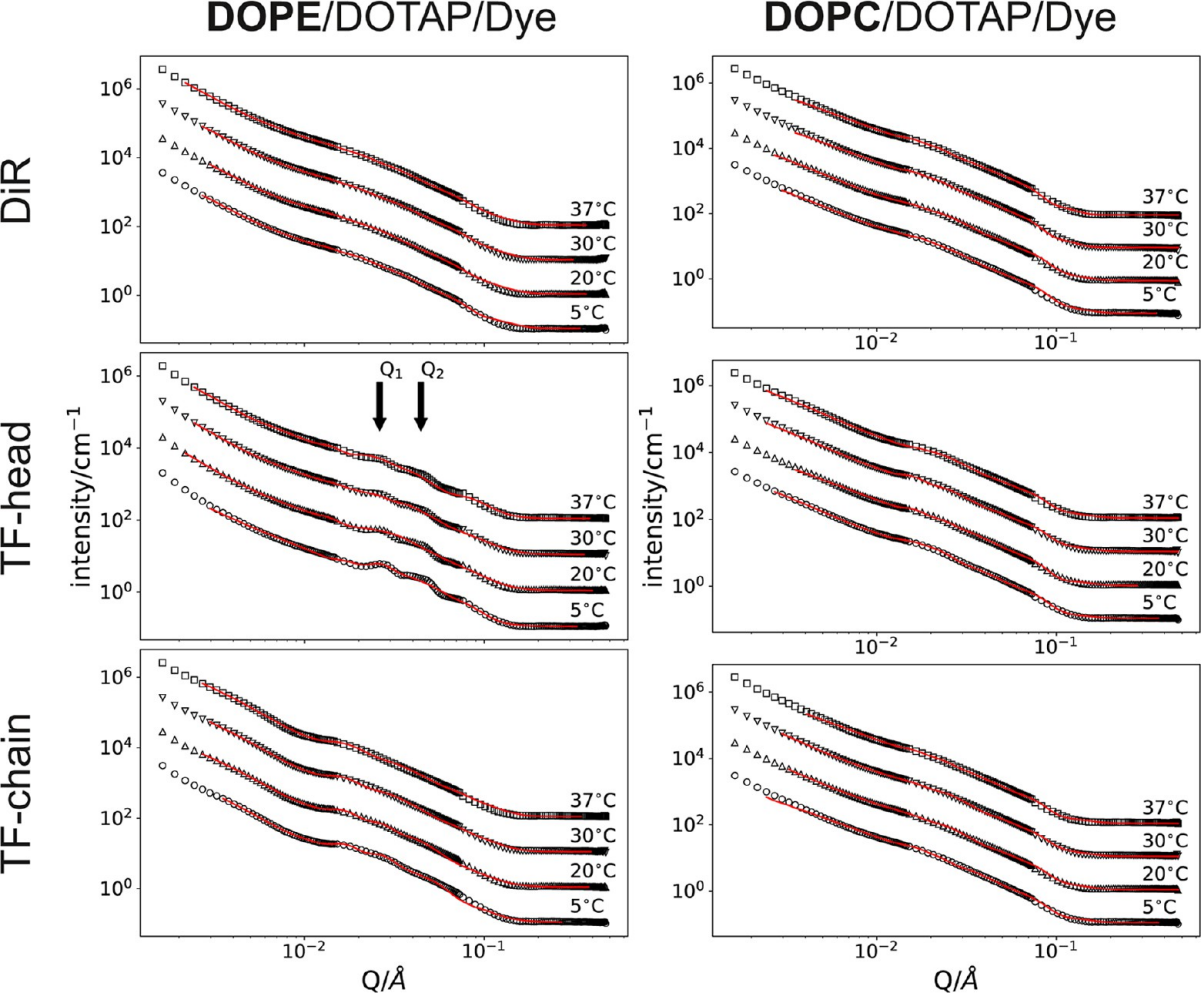
Small-angle neutron scattering curves of sFLs and sELs. DOPE/DOTAP (1/1 mol/mol) with 5% (mol/mol) of the aromatic compounds DiR, TopFluor-head (TF-head), and TopFluor-chain (TF-chain) added. Red lines indicate the fitted curves. For better visualization, curves are shifted vertically by a factor of 10 for each temperature indicated. Positions of the first and second maximum of the BCC lattice are indicated by *Q*_1_ and *Q*_2_, respectively. To compare FLs (left panel) and ELs (right panel) scattering curves, DOPE was replaced by DOPC.

Between 5 and 37 °C, there are distinct differences in the scaling factors, therefore also in the scattering contributions (Figure 6), which could be seen as a quasi-phase transition. Additionally, the decrease in the distortion parameter indicates a more extended range, and therefore, larger aggregates of BCC ordered vesicles. Thus, in general, liposomes with a higher fusogenic ability exhibit a greater amount of the BCC phase, along with a better order of the same, which in this case translates directly to domain size, akin to calculations with the Debye–Waller factor for crystals.

However, since all phases are present nearly all the time, the process would appear not to be a classical phase transition but more a shift of the most contributing phase. This finding is in agreement with the extremely small free enthalpy of phase transition inferred from earlier mechanical deformation experiments.^41^

Similar to ssNMR measurements, SANS investigations revealed a limited reproducibility in the appearance of the phase (Figure S5). Depending on thermal history, presumably the most important environmental factor, the formation of small micelles between the lipid leaflets started already at temperatures below 5 °C. In those cases, phase transition recording was not possible. We assume that sample storage at −20 °C or 4 °C before measurements influenced the nucleation process of the BCC phase more than previously expected.

The substitution of DOPE with DOPC in all analyzed mixtures led to a strongly suppressed scattering from the lamellar phase, down to virtually zero, with a diminished contribution from the BCC phase, leaving large vesicles as the dominant species in the system. Measurement curves with best fit functions overlaid are presented in Figure 6 (right panel). Indeed, in earlier experiments we had tested FL fusion efficiency with Chinese hamster ovary (CHO) cells and had found that cationic liposomes containing DOPE, here identified as samples with an increased amount of BCC phase, fused with high efficiency with the cellular plasma membrane while DOPC containing cationic liposomes, here strongly suppressed in both BCC and lamellar contribution, were far less efficient.^16^

Although FL samples were investigated at different concentrations using various techniques, all of them demonstrated the formation of a new lipid phase with increasing temperature, as well as its coexistence with the lamellar lipid phase. This latest phase exhibits numerous regions with high local curvature. Together with the high mobility measured by FRAP, these small dimensions result in fast orientation changes of the phosphate group with respect to the laboratory reference frame. The rapid molecular motions cause the appearance of a sharp peak at 0 ppm in ^31^P solid-state NMR spectra. Intriguingly, neither ssNMR nor cryo-TEM gave any indication of the appearance of the inverted hexagonal lipid phase.

We see parallels between our work and that of Siegel and co-workers who investigated the fusion mechanism of bare lipid membranes.^15,57,58^ These authors focused on the lamellar to hexagonal (*L*/*H*_II_) phase transition of *N*-methylated DOPE where in a temperature window below the proper phase transition the lamellar phase is progressively destabilized by the appearance of intermembrane contacts. They postulated two different modes of intermembrane contacts, inverted micellar intermediates and interlamellar attachments, as illustrated in Figure 7. For their systems, geometrical considerations based on molecular structure showed a core diameter of 4 nm for inverted micellar intermediates (IMI). In comparison, a size of 10–12 nm was predicted for inverted micellar intermediates (ILA).^50,59^ Because the micellar structures observed in cryo-TEM (see Figures 3, 4, and S2) exhibit sizes in the range from 10 to 20 nm, we hypothesize that these structures are also interlamellar attachments.

**Figure 7:**
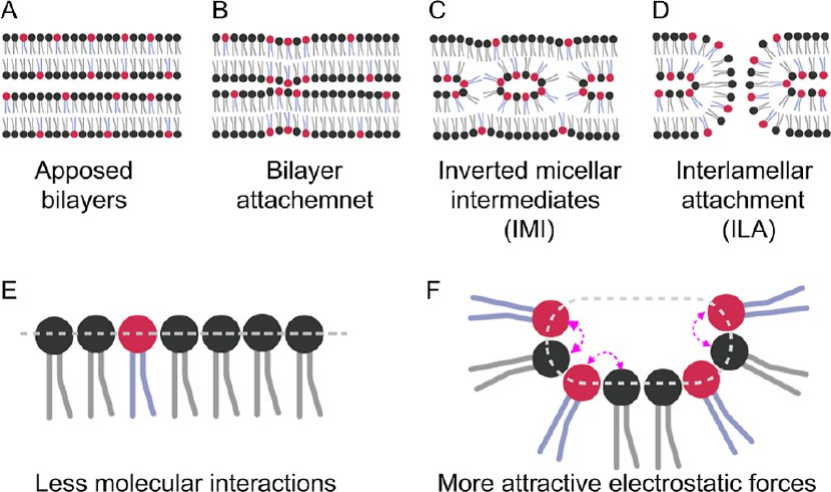
Illustration of fusion intermediates formation. (A) Two opposed bilayer membranes in close proximity to each other. (B) Local bilayer attachment can be induced by electrostatic interactions or membrane fluctuations. (C) As a consequence, micellar inclusions within the two outer membrane leaflets, so-called inverted micellar intermediates (IMI), form. (D) If IMI density is high enough, and the actual temperature is much lower than the phase transition temperature between the initial lamellar and the new 3D phase, interlamellar attachments (ILA) build up as fusion intermediate states, and the two membranes may undergo fusion. (E) Plane membrane surfaces do not favor membrane fusion processes, while (F) high curvature membrane enriched in DOPE and dye molecules allows membrane fusion intermediate formation.

Very similar structures were observed below the lamellar to hexagonal phase transition in the ternary mixture DOPE/DOPC/cholesterol.^60^ In both systems, the metastable state of the intermembrane contacts results in a strong dependence on thermal history,^61^ which we also observed. Please note that this scenario relies on the presence of a transition to a nonlamellar phase at higher temperature. For N-methylated DOPE and DOPE/DOPC/cholesterol, this high-temperature phase is hexagonal.^60^ However, bicontinuous and inverse cubic phases were also discussed in this context.^62,63^ Therefore, the fact that the nature of this postulated high-temperature phase could not be determined firmly is no major obstacle to our hypothesis.

We found clear evidence for IMI and ILA as precursors to membrane fusion. With increasing temperature, the frequency of IMI increases, and they condense locally. We hypothesize that the investigated cationic lipid mixtures containing aromatic molecules undergo similar phase transitions as described by Siegel and co-workers.^15,57,58^ If the temperature is high enough for IMI formation but does not reach the lamellar to nonlamellar phase transition temperature, fusion should be most rapid. In this state, IMI formation is adequately high. At the same time, IMIs cannot be consumed by high-temperature phase formation; instead, they contribute to ILA formation and subsequent membrane fusion.^64^ That FLs do not enter a nonlamellar high-temperature phase at the investigated temperatures underlines our hypothesis.

Moreover, the ILA-mediated fusion rate is proportional to the number of IMIs per unit area of opposed bilayers under the experimental conditions.^15^ In our case, the observed high IMI density at physiological temperature should suffice for fusion induction with biological membranes.

At this point, the question arises, which effects induce the augmented formation of fusion intermediate structures? We hypothesize that increased interfacial curvature, primarily caused by the molecular assembly of DOPE and aromatic dyes, is the most crucial factor. Due to attractive electrostatic interactions between the cationic molecular parts of DOPE, or the amine headgroup of DOTAP, and the π-electrons of the aromatics, molecular segregation becomes favorable within the bilayer (Figure 7F). The increased portion of DOPE leads to the formation of metastable bicontinuous cubic phase precursors with high curvature, embedded in a planar bilayer predominantly containing DOTAP, which forms bilayers even as a neat substance.^65^ This theory is underlined by our observation of bright fluorescence domains floating in the surrounding bilayer of GUVs (see Figure 2C,G). During fusion, the highly curved membrane structures relax, reducing bending stress, and the lipid molecules redistribute in the newly formed membrane. In this relaxed state, the original molecular composition is diluted with new lipid molecules, reducing the intermolecular interactions between cationic lipids and aromatic molecules. As a consequence, a stable membrane forms.

### Analysis of Liposomal Uptake by Neuronal Cells In Vitro

To test our hypothesis, we studied the uptake of liposomes by rat embryonic neurons. We compared FL exhibiting a high density of IMI and ILA structures with cationic liposomes lacking those intermediate structures. As shown in Figure 8, treatment of cells with FL (DOPE/DOTAP/DiR 1/1/0.1) at 37 °C resulted in fast, homogeneous, and very intense staining by the aromatic fluorescent molecule DiR (Figure 8A (upper panel), B (zoom in), and Movie S3). At increased optical magnification, a precise localization of DiR in the neuronal cellular plasma membrane was found (Figure 7, Figure 8B). Both the cell body and axons were visible. Moreover, staining was uniform, which indicated homogeneous mixing of FL and cellular membranes. Similar behavior has been observed in various cell types previously.^7^ Even FL, loaded with diverse cargo molecules, fused efficiently with cells to deliver their cargo.^8–10^ When DOPE was replaced by DOPC, the formed liposomes remained in the lamellar phase (Figures 5 and 6). Such cationic liposomes quickly adhered to the cell surface and the substrate, but fluorescence remained limited to small, less intense spots (Figure 8A and Movie S4). Probably, cells would take up some of these particles by endocytosis at a later time point. Therefore, we used the term “endocytic liposomes” or ELs to refer to them.

**Figure 8:**
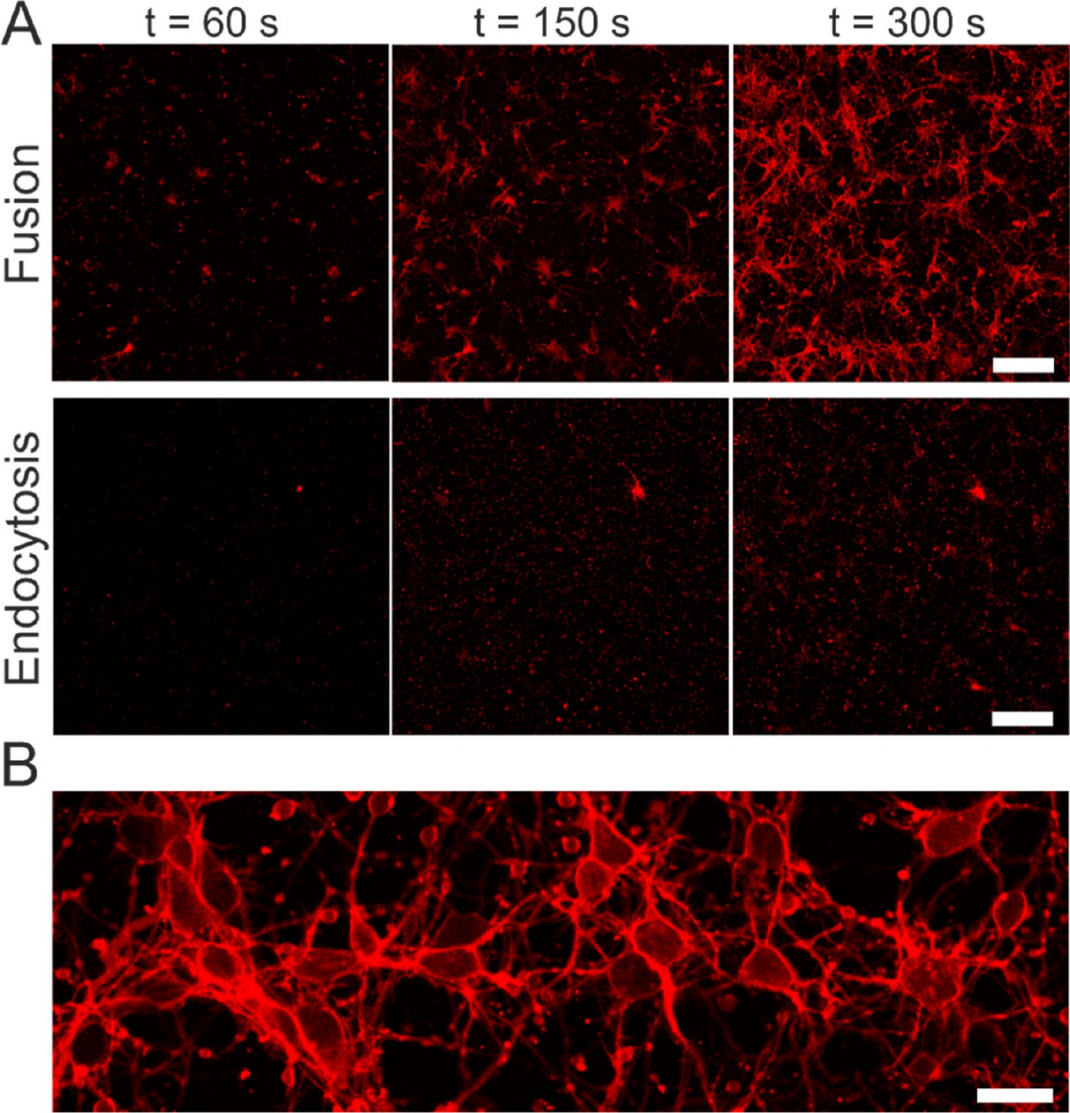
Cellular uptake of fusogenic (FL) and endocytic (EL) liposomes. (A) Rat embryonic cortical neurons were incubated with FL (DOPE/DOTAP/DiR 1/1/0.1 mol/mol; upper row) and EL (DOPC/DOTAP/DiR 1/1/0.1; lower row) for 5 min at 37 °C. Dye fluorescence (red) enabled monitoring of cellular uptake. Scale bars, 100 μm. (B) Visualization of neuronal plasma membranes, both in cell bodies and axons, upon membrane merging with FL (red). Scale bar, 20 μm.

In summary, compared to cationic liposomes, fusogenic liposomes were taken up much faster and with much higher efficiency by cells. Moreover, their molecules were distributed homogeneously over the cell surface. Our experiments on fusogenic liposomes indicate a decisive role of IMI and ILA structures in inducing fusion with the lipid bilayer membrane. However, cellular plasma membranes are covered by a thick glycocalyx. Currently, the exact mechanisms of liposomal traffic through such a dense and highly charged molecular network remain to be identified. Nevertheless, the attractive electrostatic interactions between the negatively charged glycocalix and the cationic liposomes will undouptedly play a central role. Electrostatic repulsion between cationic lipids, translational entropy, and bending energy of the highly curved structures are sufficient sources of the free energy necessary for the final fusion between membranes.

## Conclusions

Among all lipid-based approaches for drug delivery, membrane fusion is by far the most efficient method for payload insertion into mammalian cells. However, the efficient initiation of membrane fusion under physiological conditions without any protein support remained an enigma. In this and earlier works, we have demonstrated that certain cationic lipid mixtures can fuse efficiently with cell membranes. Here, the three-dimensional nanostructure of lipid particles formed from these mixtures has been resolved by several methods. We find that a high concentration of inverted micellar intermediates and interlamellar attachments accompanies high fusion efficiency. Closely related observations were made by others on DOPE liposomes at high temperature and low pH. We suggest that the same membrane structures, namely inverted micellar intermediates and interlamellar attachments, are also formed in these fusogenic lipid mixtures at body temperature and physiological pH, enabling efficient payload delivery.

## Supplementary Material





## Data Availability

Data will be made available on request.

## Notes

3D cryo-TEM tomograms of FLs and ELs. Comparison of liposomal fusion vs endocytic uptake in primary neurons. Electroswelling parameters of fusogenic GUV. Additional FRAP, cryo-TEM, ssNMR, and SANS results (PDF)
Cryo-TEM tomograms and fusion visualization (ZIP)
