# Supplementary material for: High-Density Inverted Micellar Intermediates Promote Membrane Fusion of Cationic Liposomes in Drug Delivery
Source: Langmuir. 2025 Jul 15;41(29):19055–70. doi: 10.1021/acs.langmuir.5c00659 (PMC12312148; doi:10.1021/acs.langmuir.5c00659)
Supplement: Supplementary file 1 [file la5c00659_si_001.pdf]

Supporting Information

High-density inverted micellar intermediates promote membrane fusion of cationic liposomes in drug delivery

Rejhana Kolašinac[1], Erik Strandberg[2], Laura Maria Schmitt[1], Sebastian Jaksch[3,4,5], Sabrina Berkamp[6], Georg Dreissen[1], Asma Qdemat[7], Stephan Förster[3], Carsten Sachse[6], Anne S. Ulrich[2], Rudolf Merkel[1], and Agnes Csiszár[1*]

[1]*Institute of Biological Information Processing: IBI-2 Mechanobiology, Forschungszentrum Jülich, 52428 Jülich, Germany*

[2]*Institute of Biological Interfaces: IBG-2, Karlsruher Institute of Technology, 76344 Eggenstein-Leopoldshafen, Germany*

[3]*Jülich Centre for Neutron Science: JCNS-1: Neutron Scattering and Biological Matter, Forschungszentrum Jülich, 52425 Jülich, Germany*

[4]*European Spallation Source ERIC, SE-221 00 Lund, Sweden*

[5]*Department of Physics and Astronomy, Ångström Laboratory, Uppsala University, SE-751 20 Uppsala, Sweden*

[6]*Ernst Ruska-Centre for Microscopy and Spectroscopy with Electrons: ER-C-3: Structural Biology, Forschungszentrum Jülich, 52425 Jülich, Germany*

[7]*Jülich Centre for Neutron Science: JCNS-2: Quantum Matter and Collective Phenomena, Forschungszentrum Jülich, 52425 Jülich, Germany*

*corresponding author: a.csiszar@fz-juelich.de

Table of Contents

Movies – Cryo-TEM tomograms and Fusion visualization…………………………….....S2

Tables – Characterization of Electroswelling……………………………….…………S3

Figures – Additional FRAP, cryo-TEM, NMR, and SANS results……………….…….S4-7

MOVIES

Movie S1:

Cryo-TEM tomogram and 3D structure reconstruction of DOPE/DOTAP/DiR (1/1/0.1 mol/mol) liposomes pre-incubated at 37°C before vitrification.

Movie S2:

Cryo-TEM tomogram and 3D structure reconstruction of DOPC/DOTAP/DiR (1/1/0.1 mol/mol) liposomes pre-incubated at 37°C before vitrification.

Movie S3:

Treatment of embryonic cortical neurons with the lipid mixture made of DOPE/DOTAP/DiR (1/1/0.1 mol/mol). Total imaging time was 5 min.

Movie S4:

Treatment of embryonic cortical neurons with the lipid mixture made of DOPC/DOTAP/DiR (1/1/0.1 mol/mol). Total imaging time was 8 min.

TABLES

Table S1: Parameters for electroswelling of GUVs

Protocol	Amplitude	Frequency	Duration	Temperature
Standard	1.5 V	10 Hz	1 h	21°C
Adjusted I	2.1 V	800 Hz	1 h	21°C
Adjusted II	2.1 V	800 Hz	3 h	3 °C

Table S2: Yield and average diameter of GUVs for different electroswelling protocols. Swelling procedures were repeated at least three times. For FL-GUV preparation, the lipid mixture of DOPE/DOTAP/TFhead-DOPE 1/1/0.1 mol/mol was used, while DOPC-GUVs contained TFhead-DOPE at a molar ratio of 2/0.1 mol/mol. Data are presented as mean (s.d.). The number of analyzed vesicles is indicated (N).

Sample	DOPC		FL	
Protocol	N	Diameter [µm]	N	Diameter [µm]
Standard	288	27 (6)	0	-
Adjusted I	728	15 (5)	0	-
Adjusted II	1666	10 (2)	125	8 (1)

FIGURES

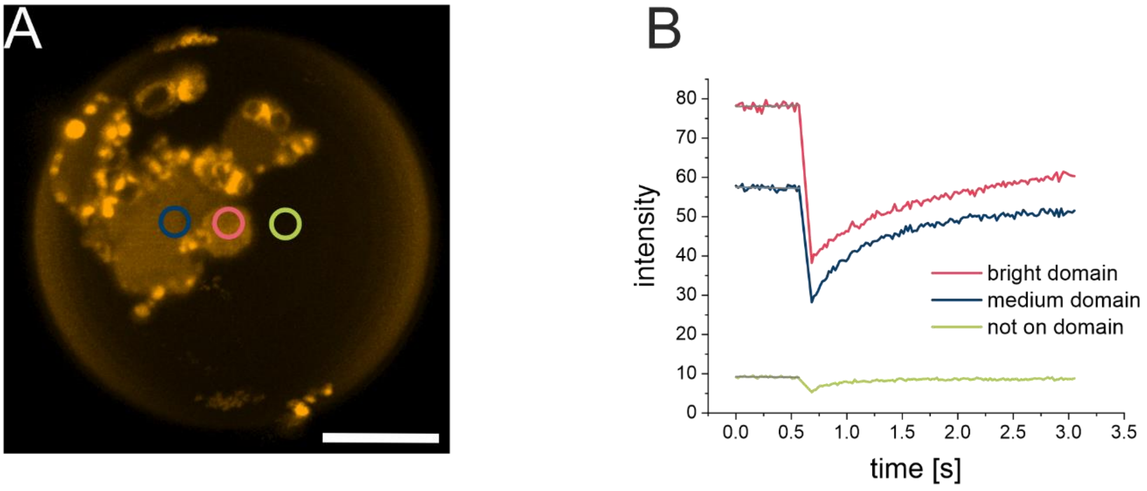

Figure S1. (A) Maximum intensity projection of an FL-GUV (DiI-orange) with regions of interest (ROIs) indicated. The same GUV as shown in Figure 1G is presented. Scale bar, 5 µm. (B) Original FRAP measurement curves within the ROIs are shown on the micrograph.

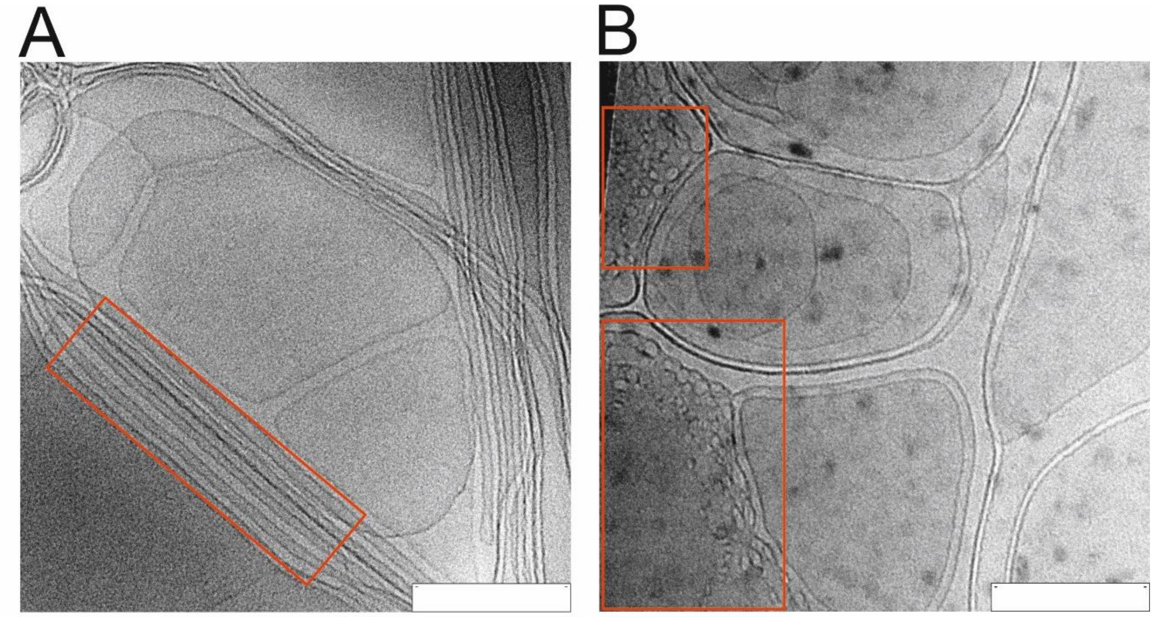

Figure S2. Cryo-TEM visualization of FLs made of DOPE/DOTAP/TF-chain (1/1/0.1 mol/mol). Phase co-existence of lamellar and isotropic phases at 20 °C (A) and 37 °C (B) was recorded. Red symbols highlight characteristic phase patterns. Scale bars, 200 nm.

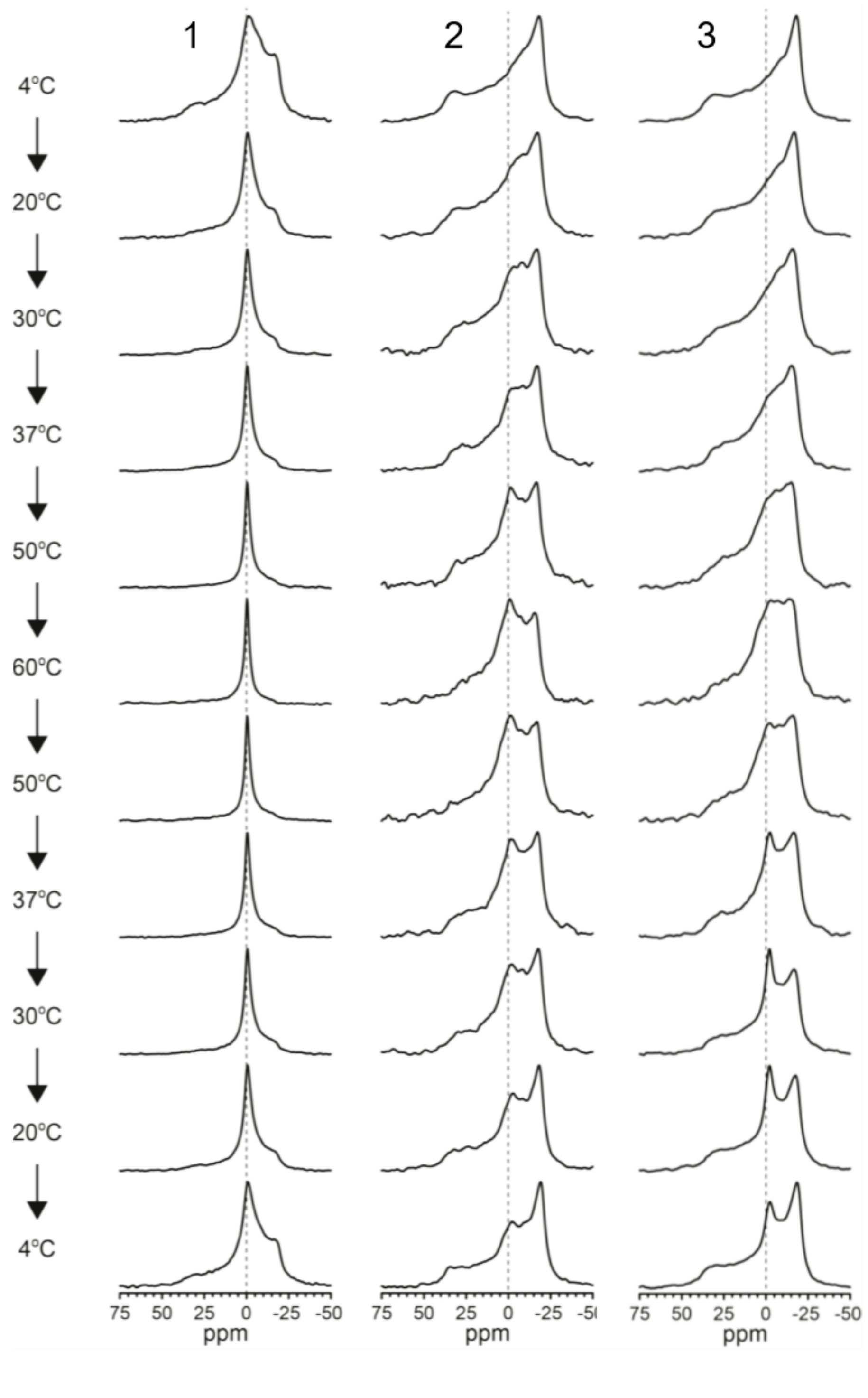

Figure S3. ^{31}P-NMR spectra of DOPE/DOTAP/TF-head (1/1/0.1 w/w) liposomes at temperatures between 4 °C and 60 °C. Three independently prepared samples were investigated. The first sample showed the simultaneous presence of lamellar and isotropic appearing phases at 4 °C. From 20 °C on, the isotropic appearing phase dominated the spectrum. The phase transition was reversible. The second sample revealed the coexistence of a lamellar phase, which is dominant at lower temperatures, and an isotropic phase, which is dominant at higher temperatures. The third sample exhibited a lamellar phase at lower temperatures. At 50 °C, an isotropic appearing phase emerged and remained upon cooling. Dashed lines mark the isotropic peak position.

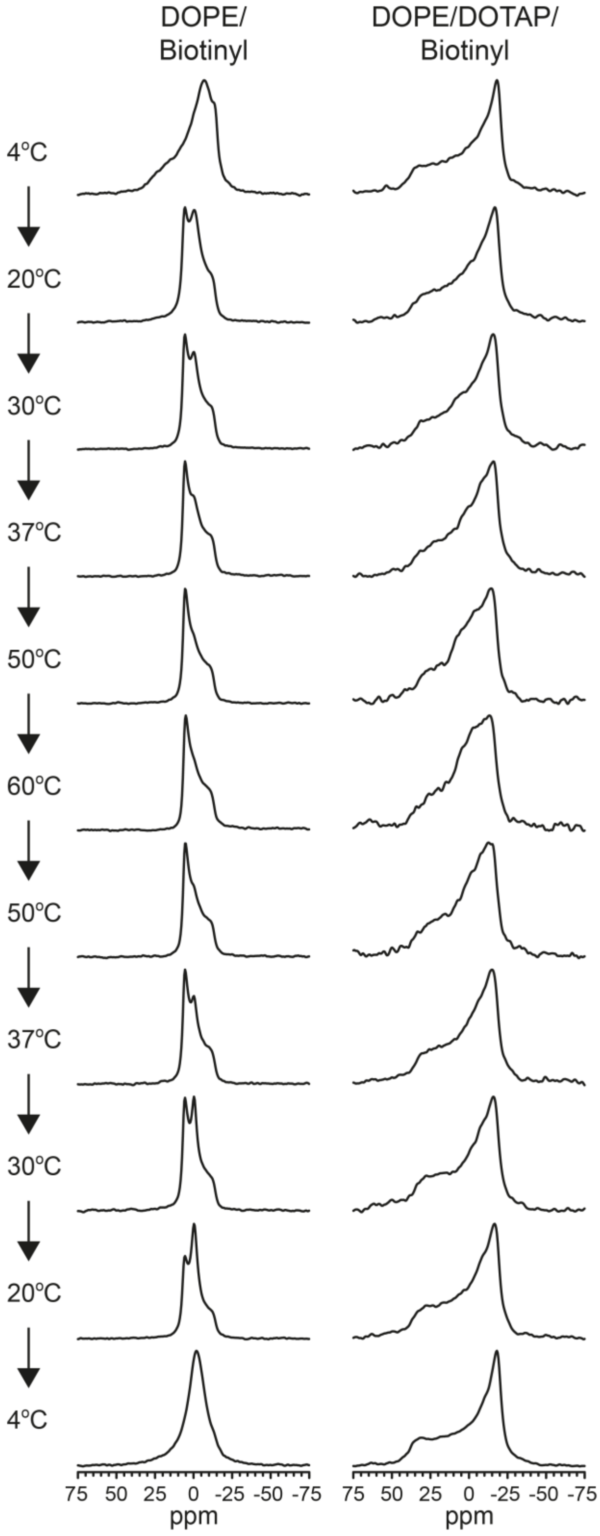

Figure S4. ^{31}P-NMR spectra of lipid systems including 5 wt% biotinyl lipids as a function of temperature. In DOPE, spectra indicate a lamellar phase with an isotropic peak at 4 °C. At 20-37 °C, the phase is hexagonal mainly, with a small isotropic peak. In DOPE/DOTAP (1/1) liposomes, the lamellar phase is present at all temperatures with an additional small, broad isotropic component above 50 °C.

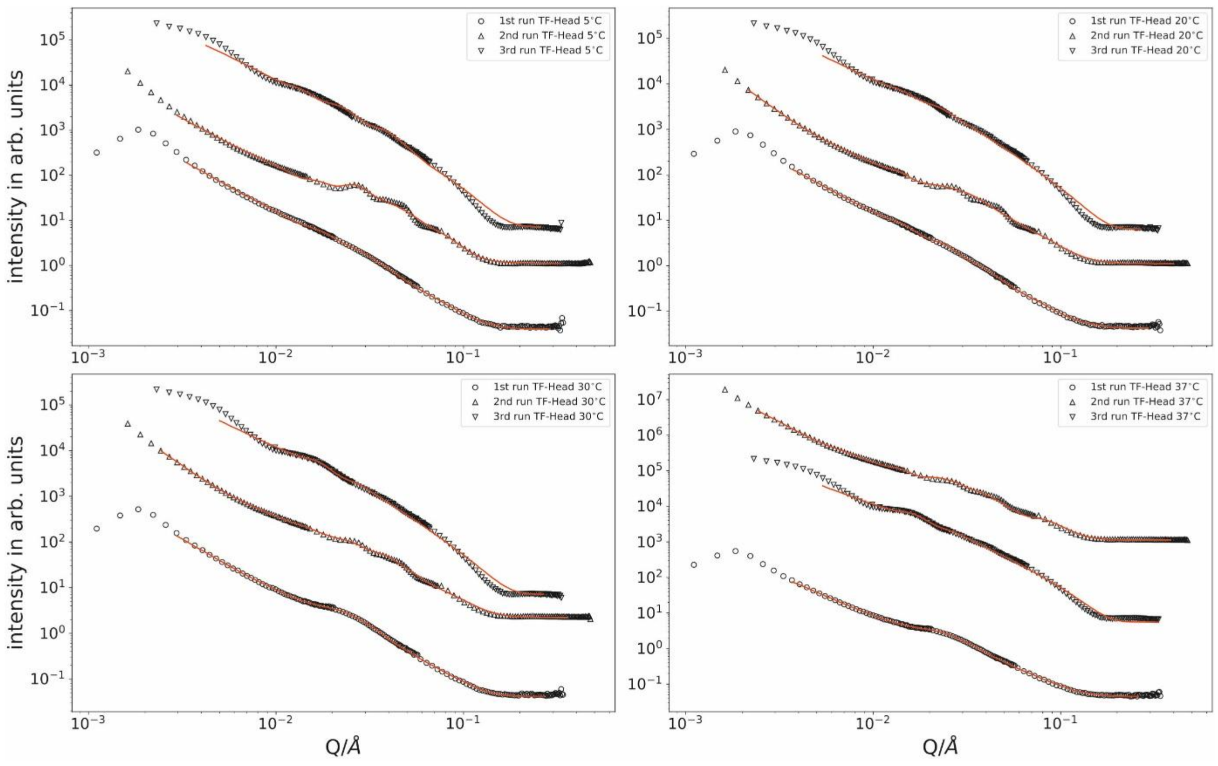

Figure S5. SANS curves of DOPE/DOTAP/TF-head (1/1/0.1 w/w) liposomes at temperatures of 5 °C, 20 °C, 30 °C, and 37 °C. Three independently prepared samples were investigated. Fit functions are shown in red.
