# Supplementary figures and images for: High-Density Inverted Micellar Intermediates Promote Membrane Fusion of Cationic Liposomes in Drug Delivery
Source: Langmuir. 2025 Jul 15;41(29):19055–70. doi: 10.1021/acs.langmuir.5c00659 (PMC12312148; doi:10.1021/acs.langmuir.5c00659)

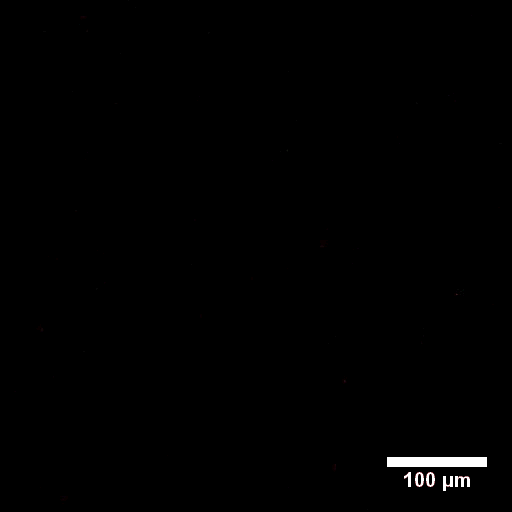

Supplement: Supplementary file 2 [file la5c00659_si_002.zip › Movie S3.gif]

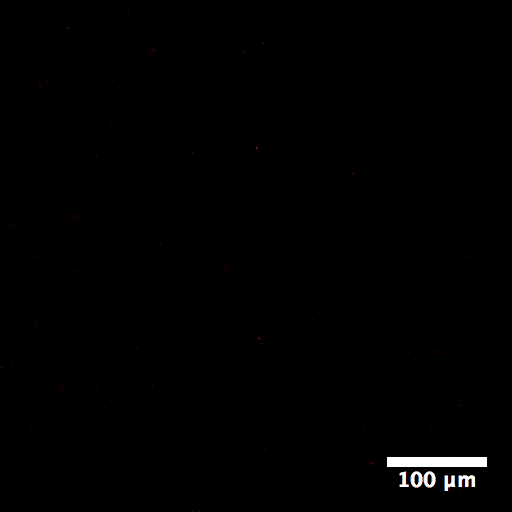

Supplement: Supplementary file 2 [file la5c00659_si_002.zip › Movie S4.gif]
